# Notice of Retraction and Replacement: Insights into undergraduate medical student selection tools: a systematic review and meta-analysis

**DOI:** 10.3352/jeehp.2024.21.41

**Published:** 2024-12-31

**Authors:** Pin-Hsiang Huang, Arash Arianpoor, Silas Taylor, Jenzel Gonzales, Boaz Shulruf

**Affiliations:** 1 Office of Medical Education, Faculty of Medicine & Health, The University of New South Wales, Sydney, Australia; 2 Department of Medical Humanities and Medical Education, College of Medicine, National Yang Ming Chiao Tung University, Taipei, Taiwan; 3 Division of Infectious Diseases, Department of Medicine, Taipei Veterans General Hospital, Taipei, Taiwan; 4 Centre for Medical and Health Sciences Education, University of Auckland, Auckland, New Zealand; The Catholic University of Korea, Korea

While continuing work on this project for another meta-analysis, the authors identified a few minor errors in the calculation of the 95% confidence intervals (CIs) for the pooled effect sizes presented in their published article, “Insights into undergraduate medical student selection tools: a systematic review and meta-analysis” [1]. These errors affected primarily the CIs, not the actual effect sizes. The authors confirmed that the issue arose from a technical error due to incorrect cell references in the Excel sheet used for the calculations. They have corrected the error and double-checked the results of their analyses using IBM SPSS Statistics ver. 28.0.1.0 (142) (IBM Corp.) and Jamovi ver. 2.6.13 (The Jamovi Project), incorporating the MAJOR (Meta-Analysis for Jamovi) 1.2.4 package, with files filtered for output.

The corrections are listed below:

­

- The number of included effect sizes has been changed from “236” to “237”, throughout the paper.

- As we consider a 95% CI not intersecting zero (i.e., P<0.05) to be significant, we have revised all instances of “P<0.01” and “P<0.001” to “P<0.05” to enhance clarity and prevent any misunderstanding.

­

- In the Abstract section, under the heading “Results”:

• Original sentences:

Overall aptitude tests predicted academic achievement in both the early and last years (0.550 & 0.371, respectively). Within aptitude tests, verbal reasoning and quantitative reasoning best predicted academic achievement in the early program (0.704 & 0.643, respectively). Neither panel interviews, multiple mini-interviews, nor situational judgement tests (SJT) yielded statistically significant pooled ES.

• Corrected sentences:

Overall aptitude tests predicted academic achievement in both the early and last years (0.550 and 0.371, respectively), as well as the end of program clinical exams (0.448). Within aptitude tests, verbal reasoning and quantitative reasoning best predicted academic achievement in the early program (0.704 and 0.643, respectively). Panel interviews showed no significant effect. However, multiple mini-interviews’ effects on early clinical exams and academic performance, as well as situational judgement tests (SJT)’s effect on early academic performance, were statistically significant.

­

- In the Methods section, under the heading “Data collection process, data items, effect measures, synthesis methods, and certainty assessment”:

• Original sentence:

This was done using Microsoft Excel (Microsoft Corp.), and confidence interval (CI) for each effect size was calculated to assess a level of certainty.

• Corrected sentences:

This was done using Microsoft Excel (Microsoft Corp.), and confidence interval (CI) for each effect size was calculated to assess a level of certainty. All analyses have been double-checked using IBM SPSS Statistics ver. 28.0.1.0 (142) (IBM Corp.) and Jamovi ver. 2.6.13 (The Jamovi Project), incorporating the MAJOR (Meta-Analysis for Jamovi) 1.2.4 package, with files filtered for output (see Dataset 1 for more details).

­

- In Table 1:

For “Aptitude testing,” the number of articles is changed from 27 to 28, and the number of effect sizes from 97 to 98.

­

- In the Results section, under the heading “Prior academic achievement”:

• Original paragraph:

Findings show that prior academic achievement best predicts academic results for early program and end of program time points with effect sizes of 0.697 (95% CI, 0.501 to 0.893) and 0.619 (95% CI, 0.509 to 0.728), respectively, as well as performance on end of program OSCE/OSLER/clinical exams (effect size, 0.545; 95% CI, 0.125 to 0.965). The effect size of prior academic achievement on early program OSCE/OSLER/clinical exams was 0.238 (95% CI, -0.106 to 0.582).

• Corrected paragraph:

Findings show that prior academic achievement best predicts academic results for early program and end of program time points with effect sizes of 0.697 (95% CI, 0.533 to 0.861) and 0.619 (95% CI, 0.533 to 0.705), respectively, as well as performance on end of program OSCE/OSLER/clinical exams (0.545; 95% CI, 0.235 to 0.855). The effect size of prior academic achievement on early program OSCE/OSLER/clinical exams was 0.238 (95% CI, 0.070 to 0.406).

­

- In the Results section, under the heading “Aptitude testing”:

• Original paragraphs:

Aptitude tests overall best predict early program and end of program academic results with the effect sizes of 0.550 (95% CI, 0.334 to 0.766) and 0.371 (95% CI, 0.219 to 0.522), respectively. Effect sizes for OSCE/OSLER/clinical exams were insignificant (early program: effect size, 0.106; 95% CI, -0.401 to 0.612; end of program: effect size, 0.448; 95% CI, -0.192 to 1.089).

With aptitude test subtest domains, abstract reasoning and verbal reasoning had effect sizes of 0.211 (95% CI, 0.001 to 0.421) and 0.305 (95% CI, 0.121 to 0.49), respectively for end of program academic results. Verbal reasoning and quantitative reasoning had effect sizes of 0.704 (95% CI, 0.426 to 0.983) and 0.643 (95% CI, 0.25 to 1.036), respectively for early program academic results.

Abstract reasoning had effect sizes below 0.2 for end of program OSCE/OSLER/clinical exams and early program academic results, with the 95% CIs intersecting zero. Interpersonal reasoning had an effect size of 0.276 for early program academic results, and verbal reasoning had an effect size of 0.298 for end of program OSCE/OSLER/clinical exams, with the 95% CIs intersecting zero. Quantitative reasoning had effect sizes of 0.216 and 0.192 for end of program OSCE/OSLER/clinical exams and academic results respectively, with the 95% CIs intersecting zero.

• Corrected paragraphs:

Aptitude tests overall best predict early program academic results (0.550; 95% CI, 0.390 to 0.710) and both end of program academic results (0.371; 95% CI, 0.278 to 0.463) and OSCE/OSLER/clinical exams (0.448; 95% CI, 0.019 to 0.877). Effect size for early program OSCE/OSLER/clinical exams was insignificant (0.106; 95% CI, -0.059 to 0.270).

With aptitude test subtest domains, abstract reasoning and verbal reasoning had effect sizes of 0.211 (95% CI, 0.117 to 0.305) and 0.305 (95% CI, 0.203 to 0.407), respectively for end of program academic results, and 0.221 (95% CI, 0.016 to 0.427) and 0.298 (95% CI, 0.004 to 0.592), respectively for end of program OSCE/OSLER/clinical exams. Moreover, verbal reasoning, quantitative reasoning, and interpersonal reasoning had effect sizes of 0.704 (95% CI, 0.471 to 0.938), 0.643 (95% CI, 0.328 to 0.958), and 0.276 (95% CI, 0.056 to 0.496), respectively for early program academic results. Although the effect of abstract reasoning for early program academic results was less than 0.2, it reached statistical significance (0.167; 95% CI, 0.009 to 0.324).

Quantitative reasoning had an effect size of 0.216 for end of program OSCE/OSLER/clinical exams, but the 95% CIs intersected zero. In contrast, while its effect size for end of program academic results was less than 0.2, it was statistically significant (0.144; 95% CI, 0.091 to 0.196).

­

- In the Results section, under the heading “Interviews”:

• Original sentence:

The effect sizes of MMI’s were 0.417 (95% CI, –0.087 to 0.921) for early program OSCE/OSLER/clinical exams and 0.195 (95% CI, –0.203 to 0.594) for early program academic results, with the 95% CIs intersecting zero.

• Corrected sentence:

The effect sizes of MMI’s were 0.417 (95% CI, 0.092 to 0.743) for early program OSCE/OSLER/clinical exams and 0.195 (95% CI, 0.019 to 0.372) for early program academic results.

­

- In the Results section, under the heading “SJT and personality testing”:

• Original sentence:

SJT had an effect size of less than 0.2 on early program academic results, with 95% CI intersecting zero.

• Corrected sentence:

While SJT had an effect size of less than 0.2 on early program academic results, its effect reached statistical significance (0.170; 95% CI, 0.032 to 0.308).

Table 2 are revised. Please see Table 2 for details of changes.

­

- In the Discussion section, third paragraph:

• Original sentences:

In this respect, when examining the remaining meta-analysis results with the number of effect sizes larger than 2 (i.e., ignoring the numbers shaded grey in Table 2 due to low evidence), there was no tool other than previous academic achievement demonstrating a statistically significant effect size (P<0.01) in predicting clinical performance outcomes. Overall aptitude testing, especially its subcategory of interpersonal reasoning, and interviews by MMI and panels may exhibit limited potential to predict OSCE with small effect sizes (between 0.2 and 0.5). Perhaps interpersonal reasoning may interact with student performance in certain stations related to patient communication skills [24], and the association between MMI and OSCE could be partially explained by the similarity in conduct of multiple stations with the grading system by checklist and global ratings [25].

• Corrected sentences:

In this respect, when examining the remaining meta-analysis results with the number of effect sizes larger than 2 (i.e., ignoring the numbers italicized in Table 2 due to low evidence), there was no tool other than previous academic achievement demonstrating a large effect size (d>0.5) in predicting clinical performance outcomes. However, it should be noted that the effects of overall aptitude testing—as well as its subcategories of abstract reasoning and verbal reasoning—and MMI were statistically significant (all P<0.05). Perhaps verbal reasoning may interact with student performance in certain stations related to patient communication skills [24], and the association between MMI and OSCE could be partially explained by the similarity in conduct of multiple stations with the grading system by checklist and global ratings [25].

­

- In the Discussion section, fourth paragraph:

• Original sentences:

First, as expected, only previous academic achievement and aptitude tests predicted academic outcomes and as expected, due to the length of the program (5–7 years), the effect drops somewhat from the early program to the end of the program. However, the specific components within the aptitude tests demonstrate that “verbal reasoning” is an important quality for success in medical school throughout the program, both in the early program and later clinical stages of curricula. On the other hand, quantitative reasoning is important in the early program, whereas abstract reasoning becomes important at the clinical stage, when significant integration of clinical, biomedical, and psychosocial information is required. Furthermore, it appears that interpersonal reasoning skills have no significant predictive value for either academic or clinical performance in the medicine program.

• Corrected sentences:

First, previous academic achievement and aptitude tests predicted academic outcomes with medium effect sizes (d>0.5) and as expected, due to the length of the program (5–7 years), the effect drops somewhat from the early program to the end of the program. In addition, the specific components within the aptitude tests demonstrate that abstract, verbal, and quantitative reasoning are important qualities for success in medical school throughout the program, both in the early program and later clinical stages of curricula. On the other hand, quantitative reasoning is important in the early program, whereas abstract and verbal reasoning becomes important at the clinical stage, when significant integration and presentation of clinical, biomedical, and psychosocial information is required. Furthermore, it appears that interpersonal reasoning skills have small, yet statistically significant predictive value for early academic performance, and more evidence is needed to understand its predictive value for clinical performance and end of program academic performance in medicine.

• Original sentences:

Second, measuring interpersonal attributes using tools that do not directly engage interpersonal interaction may not be optimal, as none of the written tests (including SJT and personality tests) measuring interpersonal interaction yielded significant effect sizes in predicting any such related outcomes. Even interviews (panel or MMI) did not effectively predict clinical or academic performance in the medicine program.

• Corrected sentences:

Second, measuring interpersonal attributes using tools that do not directly engage interpersonal interaction may not be optimal, as none of the written tests (including SJT and personality tests) measuring interpersonal interaction yielded large effect sizes (d>0.5) in predicting any such related outcomes. Although MMI predicted early clinical and academic performances and SJT predicted early academic performances with small effect sizes, more evidence is needed to determine whether they could predict clinical or academic performance at the end of medicine program.

­

- In the Discussion section, last paragraph, prior to heading “Comparison with previous studies”:

• Original sentence:

Also of note is the scarcity of empirical data about the association between SJT (with only 7 effect sizes from a single study) and/or personality tests (with only one effect size) and performance in the medicine program.

• Corrected sentence:

Also of note is the scarcity of empirical data about the association between SJT (with only 7 effect sizes from a single study and just one from another) and/or personality tests (with only one effect size) and performance in the medicine program.

­

- In the Discussion section, the first paragraph under the heading “Comparison with previous studies”:

• Original sentence:

With extensive search and calculation, our meta-analysis concluded that overall aptitude tests and 3 of its sub-categories (abstract reasoning, verbal reasoning, and quantitative reasoning) could predict academic achievement.

• Corrected sentence:

With extensive search and calculation, our meta-analysis concluded that overall aptitude tests and all its sub-categories (abstract reasoning, interpersonal reasoning, verbal reasoning, and quantitative reasoning) could predict academic achievement.

­

- In the Discussion section, the first paragraph under the heading “Implications”:

• Original sentence:

Interviews, either panel or MMI, have the potential to predict academic and clinical outcomes in the medicine program, yet more studies are required to empirically establish this finding, particularly looking at the content of the interview questions.

• Corrected sentence:

Interviews, either panel or MMI, have the potential to predict academic and clinical outcomes at the end of medicine program, yet more studies are required to empirically establish this finding, particularly looking at the content of the interview questions.

­

- In Supplement 3, table 4 under the Overall Aptitude Testing,

• Title is revised to "End of program academic results: 13 effect sizes, 11 articles"

• The following paper has been added to the table, as it was included in the calculations but was not previously listed:

­

Hendi A, Mahfouz MS, Alqassim AY, Makeen A, Somaili M, Shami MO, Alasmari AA, Darraj A, Kariri A, Ashiri A, Alhazmi AH. Admission grades as predictors of medical students’ academic performance: a cross-sectional study from Saudi Arabia. Eur J Investig Health Psychol Educ 2022;12:1572-1580. https://doi.org/10.3390/ejihpe12110110

## Figures and Tables

**Table 2.** Summary of meta-analysis results

Selection tools	OSCE/OSLER/clinical exams		Academic results		Dropout
	Early program	End of program	Early program	End of program	
Academic achievement	0.238 (-0.106 to 0.582)	**0.545 (0.125 to 0.965)**	**0.697 (0.501 to 0.893)**	**0.619 (0.509 to 0.728)**	0.205 (-0.604 to 0.540)
	**0.238 (0.070, 0.406) [9 (9)]**	**(0.235 to 0.855) [8 (7)]**	**(0.533 to 0.861) [40 (29)]**	**(0.533 to 0.705) [34 (20)]**	(–0.162 to 0.572) [3 (3)]
Aptitude tests					
Overall	0.106 (-0.401 to 0.612)	0.448 (-0.192 to 1.089)	**0.550 (0.334 to 0.766)**	**0.371 (0.219 to 0.522)**	0.425 (-0.307 to 1.157)
	(–0.059 to 0.270) [6 (5)]	**0.448 (0.019 to 0.877)** **[6 (5)]**	**(0.390 to 0.710) [20 (16)]**	**(0.278 to 0.463) [13 (11)]**	0.082 (–0.395 to 0.559) [4 (4)]
Abstract reasoning	*Nil ES*	0.128 (-0.052 to 0.309)	0.167 (-0.218 to 0.552)	**0.211 (0.001 to 0.421)**	*Nil ES*
		**0.221 (0.016 to 0.427) [4 (4)]**	**0.167 (0.009 to 0.324) [8 (6)]**	**(0.117 to 0.305) [6 (6)]**	
Interpersonal reasoning	*Nil ES*	*0.473 (0.172 to 0.773) [1 (1)]*	0.276 (-0.144 to 0.365)	*0.053 (-0.127 to 0.232)*	*Nil ES*
			**0.276 (0.056 to 0.496) [7 (5)]**	*0.074 (–0.058 to 0.206) [2 (2)]*	
Verbal reasoning	*Nil ES*	0.298 (-0.24 to 0.837)	**0.704 (0.426 to 0.983)**	**0.305 (0.121 to 0.49)**	*Nil ES*
		**0.298 (0.004 to 0.592)** **[4 (4)]**	**(0.471 to 0.938) [4 (3)]**	**(0.203 to 0.407) [3 (3)]**	
Quantitative reasoning	*Nil ES*	0.216 (-313 to 0.745)	**0.643 (0.25 to 1.036)**	0.192 (-0.321 to 0.706)	*Nil ES*
		(–0.030 to 0.462) [3 (3)]	**(0.328 to 0.958) [4 (3)]**	**0.144 (0.091 to 0.196) [3 (3)]**	
Interviews					
MMI	0.417 (-0.087 to 0.921)	*Nil ES*	0.195 (-0.203 to 0.594)	*0.229 (0.081 to 0.377) [1 (1)]*	*–0.014 (0.360 to 0.718)*
	**0.417 (0.092 to 0.743)** **[6 (5)]**		**0.195 (0.019 to 0.372)** **[4 (3)]**		*(–0.193 to 0.165) [1(1)]*
Panel	0.091 (-0.576 to 0.759)	0.372 (-0.454 to 1.197)	0.121 (-0.759 to 1)	0.135 (-0.282 to 0.552)	*0.459 (0.235 to 0.683) [1 (1)]*
	(–0.110 to 0.293) [3 (3)]	(–0.132 to 0.875) [5 (4)]	(–0.185 to 0.426) [6 (6)]	(–0.018 to 0.288) [5 (5)]	
Situational judgement tests	*Nil ES*	*Nil ES*	0.17 (-0.164 to 0.227)	*0.213 (0.044 to 0.228)*	*Nil ES*
			**0.170 (0.032 to 0.308)** **[8 (2)]**	*0.216 (0.118 to 0.313) [2 (2)]*	
Personality tests					
PQA	*0.004 (-0.104 to 0.112)*	*0.172 (-0.002 to 0.345)*	*Nil ES*	*Nil ES*	*Nil ES*
	*(–0.103 to 0.111) [1 (1)]*	*(–0.001 to 0.345) [1 (1)]*			
MMPI	*Nil ES*	*Nil ES*	*0.131 (–0.067 to 0.329) [1 (1)]*	*Nil ES*	*Nil ES*

## Supplementary materials

Supplementary files are available from Harvard Dataverse: https://doi.org/10.7910/DVN/H9NONT

Supplement 1. Retracted article with errors highlighted.

Supplement 2. Replacement article with corrections highlighted.
