# Supplementary material for: Insights into undergraduate medical student selection tools: a systematic review and meta-analysis
Source: J Educ Eval Health Prof. 2024 Dec 12;21:41. doi: 10.3352/jeehp.2024.21.22 (PMC11494217; doi:10.3352/jeehp.2024.21.22)
Supplement: Supplementary file 3 — Supplement 2. Search scripts. [file jeehp-21-22-suppl2.docx]

**Supplement 2.** Search scripts

Search was completed on 9 July 2024

Academic achievement

TITLE-ABS-KEY ("medical student" OR "medical school" OR "medical education")

AND TITLE-ABS-KEY ("selection" OR "selection tool" OR "selection algorithm" OR "admission" OR "admission tool" OR "admission criteria" OR "selection process" OR "valid*" OR "predict*")

AND TITLE-ABS-KEY ("academic achievement" OR "educational achievement" OR "educational performance" OR "prior academic" OR "academic performance" OR "high school grades" OR "high school performance")

AND TITLE-ABS-KEY ("academic record" OR "academic attainment" OR "academic performance" OR "gpa" OR "clinical assessment" OR "osce" OR "grade point average" OR "fail" OR "performance" OR "skills assessment" OR "assessment" OR "competency" OR "pbl" OR "completion")

AND PUBYEAR > 2009

- Yielded 1,060 papers

Aptitude tests

TITLE-ABS-KEY ("medical student" OR "medical school" OR "medical education") AND TITLE-ABS-KEY ("selection" OR "selection tool" OR "selection algorithm" OR "admission" OR "admission tool" OR "admission criteria" OR "selection process" OR "valid*" OR "predict*") AND TITLE-ABS-KEY ("cognitive test" OR "aptitude test" OR "UMAT" OR "undergraduate medical admissions test" OR "undergraduate medicine and health admissions test" OR "UCAT" OR "university clinical admissions test" OR "UKCAT" OR "HPAT" OR "health professions admissions test" OR "BMAT" OR "health professions admissions test") AND TITLE-ABS-KEY ("academic record" OR "academic attainment" OR "academic performance" OR "gpa" OR "clinical assessment" OR "osce" OR "grade point average" OR "fail" OR "performance" OR "skills assessment" OR "assessment" OR "competency" OR "pbl" OR "completion") AND PUBYEAR > 2009

- Yielded 188 papers

MMI and interviews

TITLE-ABS-KEY ("medical student" OR "medical school" OR "medical education")

AND TITLE-ABS-KEY ("selection" OR "selection tool" OR "selection algorithm" OR "admission" OR "admission tool" OR "admission criteria" OR "selection process")

AND TITLE-ABS-KEY ("interview" OR "multiple mini interview" OR "MMI" OR "cognitive test" OR "interview" OR "selection centre" OR "selection center")

AND TITLE-ABS-KEY ("academic record" OR "academic attainment" OR "academic performance" OR "gpa" OR "clinical assessment" OR "osce" OR "grade point average" OR "fail" OR "performance" OR "skills assessment" OR "assessment" OR "competency" OR "pbl" OR "completion")

AND PUBYEAR > 2009

- Yielded 794 papers

Situational judgement tests

TITLE-ABS-KEY ("medical student" OR "medical school" OR "medical education") AND TITLE-ABS-KEY ("selection" OR "selection tool" OR "selection algorithm" OR "admission" OR "admission tool" OR "admission criteria" OR "selection process" OR "valid*" OR "predict*") AND TITLE-ABS-KEY ("sjt" OR "situational judgement test") AND TITLE-ABS-KEY ("academic record" OR "academic attainment" OR "academic performance" OR "gpa" OR "clinical assessment" OR "osce" OR "grade point average" OR "fail" OR "performance" OR "skills assessment" OR "assessment" OR "competency" OR "pbl" OR "completion") AND PUBYEAR > 2009

- Yielded 102 papers

Personality tests

TITLE-ABS-KEY ("medical student" OR "medical school" OR "medical education")

AND TITLE-ABS-KEY ("selection" OR "selection tool" OR "selection algorithm" OR "admission" OR "admission tool" OR "admission criteria" OR "selection process" OR "valid*" OR "predict*")

AND TITLE-ABS-KEY ("personality assessment" OR "personal qualities assessment" OR "pqa")

AND TITLE-ABS-KEY ("academic record" OR "academic attainment" OR "academic performance" OR "gpa" OR "clinical assessment" OR "osce" OR "grade point average" OR "fail" OR "performance" OR "skills assessment" OR "assessment" OR "competency" OR "pbl" OR "completion")

AND PUBYEAR > 2009

- Yielded 68 papers

Combined they all yielded 2,212 papers.
