# Supplementary material for: Insights into undergraduate medical student selection tools: a systematic review and meta-analysis
Source: J Educ Eval Health Prof. 2024 Dec 12;21:41. doi: 10.3352/jeehp.2024.21.22 (PMC11494217; doi:10.3352/jeehp.2024.21.22)
Supplement: Supplementary file 4 — Supplement 3. References of articles included in the meta-analysis. [file jeehp-21-22-suppl3.docx]

**Supplement 3.** References of articles included in the meta-analysis

**-** References of articles included in the meta-analysis organized in correspondence to Table 2

**1. Academic achievement**

**a. Early objective structured clinical exam (OSCE): 9 effect sizes, 9 articles**

| No. | Article |
| --- | --- |
| 1 | Grone O, Mielke I, Knorr M, Ehrhardt M, Bergelt C. Associations between communication OSCE performance and admission interviews in medical education. Patient Educ Couns 2022;105:2270-2275. https://doi.org/10.1016/j.pec.2021.11.005 |
| 2 | Husbands A, Dowell J. Predictive validity of the Dundee multiple mini-interview. Med Educ 2013;47:717-725. https://doi.org/10.1111/medu.12193 |
| 3 | Kelly ME, Regan D, Dunne F, Henn P, Newell J, O'Flynn S. To what extent does the Health Professions Admission Test-Ireland predict performance in early undergraduate tests of communication and clinical skills?: an observational cohort study. BMC Med Educ 2013;13:68. https://doi.org/10.1186/1472-6920-13-68 |
| 4 | Krings R, Huwendiek S, Walsh N, Stricker D, Berendonk C. Predictive power of high school educational attainment and the medical aptitude test for performance during the Bachelor program in human medicine at the University of Bern: a cohort study. Swiss Med Wkly 2020;150:w20389. https://doi.org/10.4414/smw.2020.20389 |
| 5 | McManus IC, Dewberry C, Nicholson S, Dowell JS. The UKCAT-12 study: educational attainment, aptitude test performance, demographic and socio-economic contextual factors as predictors of first year outcome in a cross-sectional collaborative study of 12 UK medical schools. BMC Med 2013;11:244. https://doi.org/10.1186/1741-7015-11-244 |
| 6 | Simpson PL, Scicluna HA, Jones PD, Cole AM, O'Sullivan AJ, Harris PG, Velan G, McNeil HP. Predictive validity of a new integrated selection process for medical school admission. BMC Med Educ 2014;14:86. https://doi.org/10.1186/1472-6920-14-86 |
| 7 | Tsikas SA. Can selection interviews predict OSCE performance?: evidence from Hannover Medical School. Z Evid Fortbild Qual Gesundhwes 2022;173:85-91. https://doi.org/10.1016/j.zefq.2022.05.008 |
| 8 | Bestetti RB, Durand MT, Couto LB, Faria-Jr M, Fumagalli HF, Silva VM, Romao GS, Furlan-Daniel R, Garcia ME, Ferri SM, Reis AC, Jorge-Neto SD, Geleilete TJ. A Comparison of the academic achievement at the end of the medicine undergraduate degree program between students who only used the University Admission Test and those who used the University Admission Test Plus Marks from the High School National Exam (ENEM) at a single Brazilian center. Adv Med Educ Pract 2023;14:1185-1190. https://doi.org/10.2147/AMEP.S372822 |
| 9 | Davies DJ, Sam AH, Murphy KG, Khan SA, Choe R, Cleland J. BMAT's predictive validity for medical school performance: a retrospective cohort study. Med Educ 2022;56:936-948. https://doi.org/10.1111/medu.14819 |

**b. End of program OSCE: 8 effect sizes, 7 articles**

| No. | Article |
| --- | --- |
| 1 | Adam J, Bore M, Childs R, Dunn J, Mckendree J, Munro D, Powis D. Predictors of professional behaviour and academic outcomes in a UK medical school: a longitudinal cohort study. Med Teach 2015;37:868-880. https://doi.org/10.3109/0142159X.2015.1009023 |
| 2 | Bestetti RB, Couto LB, Roncato-Paiva P, Romão GS, Faria-Jr M, Furlan-Daniel RA, Geleilete TJM, Jorge-Neto SD, Mendonça FP, Garcia ME, Durand MT. University admission test associates with academic performance at the end of medical course in a PBL medical hybrid curriculum. Adv Med Educ Pract 2020;11:579-585. https://doi.org/10.2147/AMEP.S255732 |
| 3 | McManus IC, Woolf K, Dacre J, Paice E, Dewberry C. The Academic Backbone: longitudinal continuities in educational achievement from secondary school and medical school to MRCP(UK) and the specialist register in UK medical students and doctors. BMC Med 2013;11:242. https://doi.org/10.1186/1741-7015-11-242 |
| 4 | Poole P, Shulruf B, Rudland J, Wilkinson T. Comparison of UMAT scores and GPA in prediction of performance in medical school: a national study. Med Educ 2012;46:163-171. https://doi.org/10.1111/j.1365-2923.2011.04078.x |
| 5 | Salem RO, Al-Mously N, AlFadil S, Baalash A. Pre-admission criteria and pre-clinical achievement: Can they predict medical students performance in the clinical phase? Med Teach 2016;38 Suppl 1:S26-S30. https://doi.org/10.3109/0142159X.2016.1142511 |
| 6 | Simpson PL, Scicluna HA, Jones PD, Cole AM, O’Sullivan AJ, Harris PG, Velan G, McNeil HP. Predictive validity of a new integrated selection process for medical school admission. BMC Med Educ 2014;14:86. https://doi.org/10.1186/1472-6920-14-86 |
| 7 | Davies DJ, Sam AH, Murphy KG, Khan SA, Choe R, Cleland J. BMAT’s predictive validity for medical school performance: a retrospective cohort study. Med Educ 2022;56:936-948. https://doi.org/10.1111/medu.14819 |

**c. Early academic results: 40 effect sizes, 29 articles**

| No. | Title |
| --- | --- |
| 1 | Adeniyi OS, Araoye MA, Amali EO, Eru EU, Ojabo CO, Alao OO. Effect of using combination of O’level result with JAMB score on student performance in the first two years of medical school in Benue State University, Makurdi. Afr J Biomed Res 2010;13:189-195. |
| 2 | Al Alwan I, Al Kushi M, Tamim H, Magzoub M, Elzubeir M. Health sciences and medical college preadmission criteria and prediction of in-course academic performance: a longitudinal cohort study. Adv Health Sci Educ Theory Pract 2013;18:427-438. https://doi.org/10.1007/s10459-012-9380-1 |
| 3 | Alhadlaq AM, Alshammari OF, Alsager SM, Neel KA, Mohamed AG. Ability of admissions criteria to predict early academic performance among students of health science colleges at King Saud University, Saudi Arabia. J Dent Educ 2015;79:665-670. https://doi.org/10.1002/j.0022-0337.2015.79.6.tb05939.x |
| 4 | Alnasir FA, Jaradat AA. The effectiveness of AGU-MCAT in predicting medical student performance in year one of the College of Medicine of the Arabian Gulf University. Educ Health (Abingdon) 2011;24:447. https://doi.org/10.4103/1357-6283.101444 |
| 5 | Edwards D, Friedman T, Pearce J. Same admissions tools, different outcomes: a critical perspective on predictive validity in three undergraduate medical schools. BMC Med Educ 2013;13:173. https://doi.org/10.1186/1472-6920-13-173 |
| 6 | Fan AP, Tsai TC, Su TP, Kosik RO, Morisky DE, Chen CH, Shih WJ, Lee CH. A longitudinal study of the impact of interviews on medical school admissions in Taiwan. Eval Health Prof 2010;33:140-163. https://doi.org/10.1177/0163278710361920 |
| 7 | Gautam AP, Paudel BH, Agrawal CS, Niraula SR, Dalen JV. Examination of relationship of scores obtained in grades 10 and 12 with the entry and success in undergraduate medical education. Kathmandu Univ Med J (KUMJ) 2012;10:66-71. https://doi.org/10.3126/kumj.v10i1.6918 |
| 8 | Griffin B, Bayl-Smith P, Hu W. Predicting patterns of change and stability in student performance across a medical degree. Med Educ 2018;52:438-446. https://doi.org/10.1111/medu.13508 |
| 9 | Hewage SN, Salgado LS, Fernando GM, Liyanage PL, Pathmeswaran A, de Silva NR. Selection of medical students in Sri Lanka: time to re-think criteria? Ceylon Med J 2011;56:22-28. https://doi.org/10.4038/cmj.v56i1.2891 |
| 10 | Husbands A, Dowell J. Predictive validity of the Dundee multiple mini-interview. Med Educ 2013;47:717-725. https://doi.org/10.1111/medu.12193 |
| 11 | Irasanti SN, Akbar IB, Dewi MK, Susanti Y. The capability of selection tools to predict future academic performance of medical students. J Phys Conf Ser 2020;1469:012138. https://doi.org/10.1088/1742-6596/1469/1/012138 |
| 12 | Krings R, Huwendiek S, Walsh N, Stricker D, Berendonk C. Predictive power of high school educational attainment and the medical aptitude test for performance during the Bachelor program in human medicine at the University of Bern: a cohort study. Swiss Med Wkly 2020;150:w20389. https://doi.org/10.4414/smw.2020.20389 |
| 13 | McManus IC, Dewberry C, Nicholson S, Dowell JS. The UKCAT-12 study: educational attainment, aptitude test performance, demographic and socio-economic contextual factors as predictors of first year outcome in a cross-sectional collaborative study of 12 UK medical schools. BMC Med 2013;11:244. https://doi.org/10.1186/1741-7015-11-244 |
| 14 | McManus IC, Woolf K, Dacre J, Paice E, Dewberry C. The Academic Backbone: longitudinal continuities in educational achievement from secondary school and medical school to MRCP(UK) and the specialist register in UK medical students and doctors. BMC Med 2013;11:242. https://doi.org/10.1186/1741-7015-11-242 |
| 15 | Mercer A, Puddey IB. Admission selection criteria as predictors of outcomes in an undergraduate medical course: a prospective study. Med Teach 2011;33:997-1004. https://doi.org/10.3109/0142159X.2011.577123 |
| 16 | Mirgani Z, Shantakumari N, Hassan I. Predictors of student performance in foundation year of medical school. Sys Rev Pharm 2020;11:201-205. |
| 17 | Poole P, Shulruf B, Rudland J, Wilkinson T. Comparison of UMAT scores and GPA in prediction of performance in medical school: a national study. Med Educ 2012;46:163-171. https://doi.org/10.1111/j.1365-2923.2011.04078.x |
| 18 | Shulruf B, Poole P, Wang GY, Rudland J, Wilkinson T. How well do selection tools predict performance later in a medical programme? Adv Health Sci Educ Theory Pract 2012;17:615-626. https://doi.org/10.1007/s10459-011-9324-1 |
| 19 | Simpson PL, Scicluna HA, Jones PD, Cole AM, O’Sullivan AJ, Harris PG, Velan G, McNeil HP. Predictive validity of a new integrated selection process for medical school admission. BMC Med Educ 2014;14:86. https://doi.org/10.1186/1472-6920-14-86 |
| 20 | Nurma Y, Layli I, Kartika P, Syafarinah Nur HA. Admission assessment criteria in predicting students’ academic performance in newly established medical school. Gac Med Caracas 2021;129:S423-S428. https://doi.org/10.47307/GMC.2021.129.s2.21 |
| 21 | Almarabheh A, Shehata MH, Ismaeel A, Atwa H, Jaradat A. Predictive validity of admission criteria in predicting academic performance of medical students: a retrospective cohort study. Front Med (Lausanne) 2022;9:971926. https://doi.org/10.3389/fmed.2022.971926 |
| 22 | Althewini A, Al Baz N. Prediction of Admission Tests for medical students’ academic performance. Adv Med Educ Pract 2022;13:1287-1292. https://doi.org/10.2147/AMEP.S355474 |
| 23 | Amelung D, Zegota S, Espe L, Wittenberg T, Raupach T, Kadmon M. Considering vocational training as selection criterion for medical students: evidence for predictive validity. Adv Health Sci Educ Theory Pract 2022;27:933-948. https://doi.org/10.1007/s10459-022-10120-y |
| 24 | Bekele AT, Beza SW, Gedamu S, Berndt M. Predictors of college academic achievement for medical students: the case of Gondar University, College of Medicine and Health Sciences, Ethiopia. Adv Med Educ Pract 2023;14:603-613. https://doi.org/10.2147/AMEP.S406031 |
| 25 | Davies DJ, Sam AH, Murphy KG, Khan SA, Choe R, Cleland J. BMAT’s predictive validity for medical school performance: a retrospective cohort study. Med Educ 2022;56:936-948. https://doi.org/10.1111/medu.14819 |
| 26 | Schneid SD, Kelly CJ, Brandl K. Relationships between preadmission variables and academic outcomes for postbaccalaureate students in medical school. Adv Health Sci Educ Theory Pract 2022;27:1033-1048. https://doi.org/10.1007/s10459-022-10129-3 |
| 27 | Ballejos MP, Cestone C, Copeland HL, Dunleavy DM, Geiger T, Patel D. Predicting medical student performance with a situational judgment test in admissions. Acad Med 2024;99:175-182. https://doi.org/10.1097/ACM.0000000000005516 |
| 28 | Hendi A, Mahfouz MS, Alqassim AY, Makeen A, Somaili M, Shami MO, Names AA, Darraj A, Kariri A, Ashiri A, Alhazmi AH. Admission grades as predictors of medical students’ academic performance: a cross-sectional study from Saudi Arabia. Eur J Investig Health Psychol Educ 2022;12:1572-1580. https://doi.org/10.3390/ejihpe12110110 |
| 29 | Gebru HT, Verstegen D. Assessing predictors of students’ academic performance in Ethiopian new medical schools: a concurrent mixed-method study. BMC Med Educ 2023;23:448. https://doi.org/10.1186/s12909-023-04372-4 |

**d. End of program academic results: 34 effect sizes, 20 articles**

| No. | Article |
| --- | --- |
| 1 | Al-Rukban MO, Munshi FM, Abdulghani HM, Al-Hoqail I. The ability of the pre-admission criteria to predict performance in a Saudi medical school. Saudi Med J 2010;31:560-564. |
| 2 | Bestetti RB, Couto LB, Roncato-Paiva P, Romão GS, Faria-Jr M, Furlan-Daniel RA, Geleilete TJM, Jorge-Neto SD, Mendonça FP, Garcia ME, Durand MT. University admission test associates with academic performance at the end of medical course in a PBL medical hybrid curriculum. Adv Med Educ Pract 2020;11:579-585. https://doi.org/10.2147/AMEP.S255732 |
| 3 | Bußenius L, Harendza S. Are different medical school admission tests associated with the outcomes of a simulation-based OSCE? BMC Med Educ 2021;21:263. https://doi.org/10.1186/s12909-021-02703-x |
| 4 | Fan AP, Tsai TC, Su TP, Kosik RO, Morisky DE, Chen CH, Shih WJ, Lee CH. A longitudinal study of the impact of interviews on medical school admissions in Taiwan. Eval Health Prof 2010;33:140-163. https://doi.org/10.1177/0163278710361920 |
| 5 | Gautam AP, Paudel BH, Agrawal CS, Niraula SR, Dalen JV. Examination of relationship of scores obtained in grades 10 and 12 with the entry and success in undergraduate medical education. Kathmandu Univ Med J (KUMJ) 2012;10:66-71. https://doi.org/10.3126/kumj.v10i1.6918 |
| 6 | Guraya SY. High school grades are not reliable predictors of academic performance in undergraduate medical school: a study from a Saudi medical school. Biomed Pharmacol J 2015;5:219-225. http://biomedpharmajournal.org/?p=2475 |
| 7 | Maslov Kruzicevic S, Barisic KJ, Banozic A, Esteban CD, Sapunar D, Puljak L. Predictors of attrition and academic success of medical students: a 30-year retrospective study. PLoS One 2012;7:e39144. https://doi.org/10.1371/journal.pone.0039144 |
| 8 | McManus IC, Woolf K, Harrison D, Tiffin PA, Paton LW, Cheung KY, Smith DT. Predictive validity of A-level grades and teacher-predicted grades in UK medical school applicants: a retrospective analysis of administrative data in a time of COVID-19. BMJ Open 2021;11:e047354. https://doi.org/10.1136/bmjopen-2020-047354 |
| 9 | McManus IC, Woolf K, Dacre J, Paice E, Dewberry C. The Academic Backbone: longitudinal continuities in educational achievement from secondary school and medical school to MRCP(UK) and the specialist register in UK medical students and doctors. BMC Med 2013;11:242. https://doi.org/10.1186/1741-7015-11-242 |
| 10 | Mercer A, Puddey IB. Admission selection criteria as predictors of outcomes in an undergraduate medical course: a prospective study. Med Teach 2011;33:997-1004. https://doi.org/10.3109/0142159X.2011.577123 |
| 11 | Meyer H, Zimmermann S, Hissbach J, Klusmann D, Hampe W. Selection and academic success of medical students in Hamburg, Germany. BMC Med Educ 2019;19:23. https://doi.org/10.1186/s12909-018-1443-4 |
| 12 | Poole P, Shulruf B, Rudland J, Wilkinson T. Comparison of UMAT scores and GPA in prediction of performance in medical school: a national study. Med Educ 2012;46:163-171. https://doi.org/10.1111/j.1365-2923.2011.04078.x |
| 13 | Simpson PL, Scicluna HA, Jones PD, Cole AM, O’Sullivan AJ, Harris PG, Velan G, McNeil HP. Predictive validity of a new integrated selection process for medical school admission. BMC Med Educ 2014;14:86. https://doi.org/10.1186/1472-6920-14-86 |
| 14 | Almarabheh A, Shehata MH, Ismaeel A, Atwa H, Jaradat A. Predictive validity of admission criteria in predicting academic performance of medical students: a retrospective cohort study. Front Med (Lausanne) 2022;9:971926. https://doi.org/10.3389/fmed.2022.971926 |
| 15 | Davies DJ, Sam AH, Murphy KG, Khan SA, Choe R, Cleland J. BMAT's predictive validity for medical school performance: a retrospective cohort study. Med Educ 2022;56:936-948. https://doi.org/10.1111/medu.14819 |
| 16 | Hendi A, Mahfouz MS, Alqassim AY, Makeen A, Somaili M, Shami MO, Names AA, Darraj A, Kariri A, Ashiri A, Alhazmi AH. Admission grades as predictors of medical students’ academic performance: a cross-sectional study from Saudi Arabia. Eur J Investig Health Psychol Educ 2022;12:1572-1580. https://doi.org/10.3390/ejihpe12110110 |
| 17 | Ozeki S, Kasamo S, Inoue H, Matsumoto S. Does regional quota status affect the performance of undergraduate medical students in Japan?: a 10-year analysis. Int J Med Educ 2022;13:307-314. https://doi.org/10.5116/ijme.6372.1fce |
| 18 | Tamimi A, Hassuneh M, Tamimi I, Juweid M, Shibli D, AlMasri B, Tamimi F. Admission criteria and academic performance in medical school. BMC Med Educ 2023;23:273. https://doi.org/10.1186/s12909-023-04251-y |
| 19 | Bekele AT, Beza SW, Gedamu S, Berndt M. Predictors of college academic achievement for medical students: the case of Gondar University, College of Medicine and Health Sciences, Ethiopia. Adv Med Educ Pract 2023;14:603-613. https://doi.org/10.2147/AMEP.S406031 |
| 20 | Zuljevic MF, Buljan I. Academic and non-academic predictors of academic performance in medical school: an exploratory cohort study. BMC Med Educ 2022;22:366. https://doi.org/10.1186/s12909-022-03436-1 |

**e. Dropout: 3 effect sizes, 3 articles**

| No. | Title |
| --- | --- |
| 1 | Kadmon G, Resch F, Duelli R, Kadmon M. Predictive value of the school-leaving grade and prognosis of different admission groups for academic performance and continuity in the medical course: a longitudinal study. GMS Z Med Ausbild 2014;31:Doc21. https://doi.org/10.3205/zma000913 |
| 2 | Shulruf B, Poole P, Wang GY, Rudland J, Wilkinson T. How well do selection tools predict performance later in a medical programme? Adv Health Sci Educ Theory Pract 2012;17:615-626. https://doi.org/10.1007/s10459-011-9324-1 |
| 3 | Hefny AF, Almansoori TM, El-Zubeir M, AlBawardi A, Shaban S, Magzoub ME, Zoubeidi T, Mansour NA. Relationship between admission selection tools and student attrition in the early years of medical school. J Taibah Univ Med Sci 2024;19:447-452. https://doi.org/10.1016/j.jtumed.2024.02.004 |

**2. Aptitude testing**

**a. Overall**

*1) Early OSCE: 6 effect sizes, 5 articles*

| No. | Title |
| --- | --- |
| 1 | Husbands A, Dowell J. Predictive validity of the Dundee multiple mini-interview. Med Educ 2013;47:717-725. https://doi.org/10.1111/medu.12193 |
| 2 | Kelly ME, Regan D, Dunne F, Henn P, Newell J, O’Flynn S. To what extent does the Health Professions Admission Test-Ireland predict performance in early undergraduate tests of communication and clinical skills?: an observational cohort study. BMC Med Educ 2013;13:68. https://doi.org/10.1186/1472-6920-13-68 |
| 3 | Krings R, Huwendiek S, Walsh N, Stricker D, Berendonk C. Predictive power of high school educational attainment and the medical aptitude test for performance during the Bachelor program in human medicine at the University of Bern: a cohort study. Swiss Med Wkly 2020;150:w20389. https://doi.org/10.4414/smw.2020.20389 |
| 4 | Simpson PL, Scicluna HA, Jones PD, Cole AM, O’Sullivan AJ, Harris PG, Velan G, McNeil HP. Predictive validity of a new integrated selection process for medical school admission. BMC Med Educ 2014;14:86. https://doi.org/10.1186/1472-6920-14-86 |
| 5 | Yates J, James D. The value of the UK Clinical Aptitude Test in predicting pre-clinical performance: a prospective cohort study at Nottingham Medical School. BMC Med Educ 2010;10:55. https://doi.org/10.1186/1472-6920-10-55 |

*2) End of program OSCE: 6 effect sizes, 5 articles*

| No. | Title |
| --- | --- |
| 1 | Husbands A, Dowell J. Predictive validity of the Dundee multiple mini-interview. Med Educ 2013;47:717-725. https://doi.org/10.1111/medu.12193 |
| 2 | Kelly ME, Regan D, Dunne F, Henn P, Newell J, O'Flynn S. To what extent does the Health Professions Admission Test-Ireland predict performance in early undergraduate tests of communication and clinical skills?: an observational cohort study. BMC Med Educ 2013;13:68. https://doi.org/10.1186/1472-6920-13-68 |
| 3 | Krings R, Huwendiek S, Walsh N, Stricker D, Berendonk C. Predictive power of high school educational attainment and the medical aptitude test for performance during the Bachelor program in human medicine at the University of Bern: a cohort study. Swiss Med Wkly 2020;150:w20389. https://doi.org/10.4414/smw.2020.20389 |
| 4 | Simpson PL, Scicluna HA, Jones PD, Cole AM, O’Sullivan AJ, Harris PG, Velan G, McNeil HP. Predictive validity of a new integrated selection process for medical school admission. BMC Med Educ 2014;14:86. https://doi.org/10.1186/1472-6920-14-86 |
| 5 | Yates J, James D. The value of the UK Clinical Aptitude Test in predicting pre-clinical performance: a prospective cohort study at Nottingham Medical School. BMC Med Educ 2010;10:55. https://doi.org/10.1186/1472-6920-10-55 |

*3) Early academic results: 20 effect sizes, 16 articles*

| No. | Title |
| --- | --- |
| 1 | Adam J, Bore M, McKendree J, Munro D, Powis D. Can personal qualities of medical students predict in-course examination success and professional behaviour?: an exploratory prospective cohort study. BMC Med Educ 2012;12:69. https://doi.org/10.1186/1472-6920-12-69 |
| 2 | Al Alwan I, Al Kushi M, Tamim H, Magzoub M, Elzubeir M. Health sciences and medical college preadmission criteria and prediction of in-course academic performance: a longitudinal cohort study. Adv Health Sci Educ Theory Pract 2013;18:427-438. https://doi.org/10.1007/s10459-012-9380-1 |
| 3 | Cerutti B, Bernheim L, van Gessel E. The predictive validity of the aptitude test for the performance of students starting a medical curriculum. Swiss Med Wkly 2013;143:w13872. https://doi.org/10.4414/smw.2013.13872 |
| 4 | Edwards D, Friedman T, Pearce J. Same admissions tools, different outcomes: a critical perspective on predictive validity in three undergraduate medical schools. BMC Med Educ 2013;13:173. https://doi.org/10.1186/1472-6920-13-173 |
| 5 | Griffin B, Bayl-Smith P, Hu W. Predicting patterns of change and stability in student performance across a medical degree. Med Educ 2018;52:438-446. https://doi.org/10.1111/medu.13508 |
| 6 | Husbands A, Dowell J. Predictive validity of the Dundee multiple mini-interview. Med Educ 2013;47:717-725. https://doi.org/10.1111/medu.12193 |
| 7 | Krings R, Huwendiek S, Walsh N, Stricker D, Berendonk C. Predictive power of high school educational attainment and the medical aptitude test for performance during the Bachelor program in human medicine at the University of Bern: a cohort study. Swiss Med Wkly 2020;150:w20389. https://doi.org/10.4414/smw.2020.20389 |
| 8 | Lievens F. Adjusting medical school admission: assessing interpersonal skills using situational judgement tests. Med Educ 2013;47:182-189. https://doi.org/10.1111/medu.12089 |
| 9 | Mercer A, Puddey IB. Admission selection criteria as predictors of outcomes in an undergraduate medical course: a prospective study. Med Teach 2011;33:997-1004. https://doi.org/10.3109/0142159X.2011.577123 |
| 10 | Poole P, Shulruf B, Rudland J, Wilkinson T. Comparison of UMAT scores and GPA in prediction of performance in medical school: a national study. Med Educ 2012;46:163-171. https://doi.org/10.1111/j.1365-2923.2011.04078.x |
| 11 | Shulruf B, Poole P, Wang GY, Rudland J, Wilkinson T. How well do selection tools predict performance later in a medical programme? Adv Health Sci Educ Theory Pract 2012;17:615-626. https://doi.org/10.1007/s10459-011-9324-1 |
| 12 | Simpson PL, Scicluna HA, Jones PD, Cole AM, O’Sullivan AJ, Harris PG, Velan G, McNeil HP. Predictive validity of a new integrated selection process for medical school admission. BMC Med Educ 2014;14:86. https://doi.org/10.1186/1472-6920-14-86 |
| 13 | Yates J, James D. The value of the UK Clinical Aptitude Test in predicting pre-clinical performance: a prospective cohort study at Nottingham Medical School. BMC Med Educ 2010;10:55. https://doi.org/10.1186/1472-6920-10-55 |
| 14 | Althewini A, Al Baz N. Prediction of Admission Tests for medical students’ academic performance. Adv Med Educ Pract 2022;13:1287-1292. https://doi.org/10.2147/AMEP.S355474 |
| 15 | Amelung D, Zegota S, Espe L, Wittenberg T, Raupach T, Kadmon M. Considering vocational training as selection criterion for medical students: evidence for predictive validity. Adv Health Sci Educ Theory Pract 2022;27:933-948. https://doi.org/10.1007/s10459-022-10120-y |
| 16 | Hendi A, Mahfouz MS, Alqassim AY, Makeen A, Somaili M, Shami MO, Names AA, Darraj A, Kariri A, Ashiri A, Alhazmi AH. Admission grades as predictors of medical students’ academic performance: a cross-sectional study from Saudi Arabia. Eur J Investig Health Psychol Educ 2022;12:1572-1580. https://doi.org/10.3390/ejihpe12110110 |

*4) End of program academic results: 13 effect sizes, 11 articles*

| No. | Title |
| --- | --- |
| 1 | Albishri JA, Aly SM, Alnemary Y. Admission criteria to Saudi medical schools: which is the best predictor for successful achievement? Saudi Med J 2012;33:1222-1226. |
| 2 | Hendi A, Mahfouz MS, Alqassim AY, Makeen A, Somaili M, Shami MO, Names AA, Darraj A, Kariri A, Ashiri A, Alhazmi AH. Admission grades as predictors of medical students’ academic performance: a cross-sectional study from Saudi Arabia. Eur J Investig Health Psychol Educ 2022;12:1572-1580. https://doi.org/10.3390/ejihpe12110110 |
| 3 | Lievens F. Adjusting medical school admission: assessing interpersonal skills using situational judgement tests. Med Educ 2013;47:182-189. https://doi.org/10.1111/medu.12089 |
| 4 | MacKenzie RK, Cleland JA, Ayansina D, Nicholson S. Does the UKCAT predict performance on exit from medical school?: a national cohort study. BMJ Open 2016;6:e011313. https://doi.org/10.1136/bmjopen-2016-011313 |
| 5 | McManus IC, Dewberry C, Nicholson S, Dowell JS. The UKCAT-12 study: educational attainment, aptitude test performance, demographic and socio-economic contextual factors as predictors of first year outcome in a cross-sectional collaborative study of 12 UK medical schools. BMC Med 2013;11:244. https://doi.org/10.1186/1741-7015-11-244 |
| 6 | McManus IC, Woolf K, Harrison D, Tiffin PA, Paton LW, Cheung KYF, Smith DT. Predictive validity of A-level grades and teacher-predicted grades in UK medical school applicants: a retrospective analysis of administrative data in a time of COVID-19. BMJ Open 2021;11:e047354. https://doi.org/10.1136/bmjopen-2020-047354 |
| 7 | Mercer A, Puddey IB. Admission selection criteria as predictors of outcomes in an undergraduate medical course: a prospective study. Med Teach 2011;33:997-1004. https://doi.org/10.3109/0142159X.2011.577123 |
| 8 | Poole P, Shulruf B, Rudland J, Wilkinson T. Comparison of UMAT scores and GPA in prediction of performance in medical school: a national study. Med Educ 2012;46:163-171. https://doi.org/10.1111/j.1365-2923.2011.04078.x |
| 9 | Simpson PL, Scicluna HA, Jones PD, Cole AM, O’Sullivan AJ, Harris PG, Velan G, McNeil HP. Predictive validity of a new integrated selection process for medical school admission. BMC Med Educ 2014;14:86. https://doi.org/10.1186/1472-6920-14-86 |
| 10 | Wilkinson D, Zhang J, Parker M. Predictive validity of the Undergraduate Medicine and Health Sciences Admission Test for medical students’ academic performance. Med J Aust 2011;194:341-344. https://doi.org/10.5694/j.1326-5377.2011.tb03002.x |
| 11 | Yates J, James D. The UK Clinical Aptitude Test and clinical course performance at Nottingham: a prospective cohort study. BMC Med Educ 2013;13:32. https://doi.org/10.1186/1472-6920-13-32 |

*5) Dropout: 4 effect sizes, 4 articles*

| No. | Title |
| --- | --- |
| 1 | Kadmon G, Kadmon M. Academic performance of students with the highest and mediocre school-leaving grades: does the aptitude test for medical studies (TMS) balance their prognoses? GMS J Med Educ 2016;33:Doc7. https://doi.org/10.3205/zma001006 |
| 2 | Kraft HG, Lamina C, Kluckner T, Wild C, Prodinger WM. Paradise lost or paradise regained?: changes in admission system affect academic performance and drop-out rates of medical students. Med Teach 2013;35:e1123-e1129. https://doi.org/10.3109/0142159X.2012.733835 |
| 3 | Shulruf B, Poole P, Wang GY, Rudland J, Wilkinson T. How well do selection tools predict performance later in a medical programme? Adv Health Sci Educ Theory Pract 2012;17:615-626. https://doi.org/10.1007/s10459-011-9324-1 |
| 4 | Abbiati M, Cerutti B. Do students’ personality traits change during medical training?: a longitudinal cohort study. Adv Health Sci Educ Theory Pract 2023;28:1079-1092. https://doi.org/10.1007/s10459-023-10205-2 |

***b. Abstract reasoning***

*1) Early OSCE: nil*

*2) End of program OSCE: 4 effect sizes, 4 articles*

| No. | Article |
| --- | --- |
| 1 | Adam J, Bore M, Childs R, Dunn J, Mckendree J, Munro D, Powis D. Predictors of professional behaviour and academic outcomes in a UK medical school: a longitudinal cohort study. Med Teach 2015;37:868-880. https://doi.org/10.3109/0142159X.2015.1009023 |
| 2 | Kelly ME, Regan D, Dunne F, Henn P, Newell J, O’Flynn S. To what extent does the Health Professions Admission Test-Ireland predict performance in early undergraduate tests of communication and clinical skills?: an observational cohort study. BMC Med Educ 2013;13:68. https://doi.org/10.1186/1472-6920-13-68 |
| 3 | McManus IC, Dewberry C, Nicholson S, Dowell JS. The UKCAT-12 study: educational attainment, aptitude test performance, demographic and socio-economic contextual factors as predictors of first year outcome in a cross-sectional collaborative study of 12 UK medical schools. BMC Med 2013;11:244. https://doi.org/10.1186/1741-7015-11-244 |
| 4 | Yates J, James D. The value of the UK Clinical Aptitude Test in predicting pre-clinical performance: a prospective cohort study at Nottingham Medical School. BMC Med Educ 2010;10:55. https://doi.org/10.1186/1472-6920-10-55 |

*3) Early academic results: 8 effect sizes, 6 articles*

| No. | Article |
| --- | --- |
| 1 | Adam J, Bore M, McKendree J, Munro D, Powis D. Can personal qualities of medical students predict in-course examination success and professional behaviour?: an exploratory prospective cohort study. BMC Med Educ 2012;12:69. https://doi.org/10.1186/1472-6920-12-69 |
| 2 | Edwards D, Friedman T, Pearce J. Same admissions tools, different outcomes: a critical perspective on predictive validity in three undergraduate medical schools. BMC Med Educ 2013;13:173. https://doi.org/10.1186/1472-6920-13-173 |
| 3 | Griffin B, Bayl-Smith P, Hu W. Predicting patterns of change and stability in student performance across a medical degree. Med Educ 2018;52:438-446. https://doi.org/10.1111/medu.13508 |
| 4 | Mercer A, Puddey IB. Admission selection criteria as predictors of outcomes in an undergraduate medical course: a prospective study. Med Teach 2011;33:997-1004. https://doi.org/10.3109/0142159X.2011.577123 |
| 5 | Shulruf B, Poole P, Wang GY, Rudland J, Wilkinson T. How well do selection tools predict performance later in a medical programme? Adv Health Sci Educ Theory Pract 2012;17:615-626. https://doi.org/10.1007/s10459-011-9324-1 |
| 6 | Yates J, James D. The value of the UK Clinical Aptitude Test in predicting pre-clinical performance: a prospective cohort study at Nottingham Medical School. BMC Med Educ 2010;10:55. https://doi.org/10.1186/1472-6920-10-55 |

*4) End of program academic results: 6 effect sizes, 6 articles*

| No. | Article |
| --- | --- |
| 1 | Adam J, Bore M, Childs R, Dunn J, Mckendree J, Munro D, Powis D. Predictors of professional behaviour and academic outcomes in a UK medical school: a longitudinal cohort study. Med Teach 2015;37:868-880. https://doi.org/10.3109/0142159X.2015.1009023 |
| 2 | MacKenzie RK, Cleland JA, Ayansina D, Nicholson S. Does the UKCAT predict performance on exit from medical school?: a national cohort study. BMJ Open 2016;6:e011313. https://doi.org/10.1136/bmjopen-2016-011313 |
| 3 | McManus IC, Dewberry C, Nicholson S, Dowell JS. The UKCAT-12 study: educational attainment, aptitude test performance, demographic and socio-economic contextual factors as predictors of first year outcome in a cross-sectional collaborative study of 12 UK medical schools. BMC Med 2013;11:244. https://doi.org/10.1186/1741-7015-11-244 |
| 4 | Mercer A, Puddey IB. Admission selection criteria as predictors of outcomes in an undergraduate medical course: a prospective study. Med Teach 2011;33:997-1004. https://doi.org/10.3109/0142159X.2011.577123 |
| 5 | Wilkinson D, Zhang J, Parker M. Predictive validity of the Undergraduate Medicine and Health Sciences Admission Test for medical students’ academic performance. Med J Aust 2011;194:341-344. https://doi.org/10.5694/j.1326-5377.2011.tb03002.x |
| 6 | Yates J, James D. The UK Clinical Aptitude Test and clinical course performance at Nottingham: a prospective cohort study. BMC Med Educ 2013;13:32. https://doi.org/10.1186/1472-6920-13-32 |

*5) Dropout: nil*

***c. Interpersonal reasoning***

*1) Early OSCE: nil*

*2) End of program OSCE: 1 effect size, 1 article*

| No. | Article |
| --- | --- |
| 1 | Kelly ME, Regan D, Dunne F, Henn P, Newell J, O'Flynn S. To what extent does the Health Professions Admission Test-Ireland predict performance in early undergraduate tests of communication and clinical skills?: an observational cohort study. BMC Med Educ 2013;13:68. https://doi.org/10.1186/1472-6920-13-68 |

*3) Early academic results: 7 effect sizes, 5 articles*

| No. | Article |
| --- | --- |
| 1 | Edwards D, Friedman T, Pearce J. Same admissions tools, different outcomes: a critical perspective on predictive validity in three undergraduate medical schools. BMC Med Educ 2013;13:173. https://doi.org/10.1186/1472-6920-13-173 |
| 2 | Griffin B, Bayl-Smith P, Hu W. Predicting patterns of change and stability in student performance across a medical degree. Med Educ 2018;52:438-446. https://doi.org/10.1111/medu.13508 |
| 3 | Kelly ME, Regan D, Dunne F, Henn P, Newell J, O’Flynn S. To what extent does the Health Professions Admission Test-Ireland predict performance in early undergraduate tests of communication and clinical skills?: an observational cohort study. BMC Med Educ 2013;13:68. https://doi.org/10.1186/1472-6920-13-68 |
| 4 | Mercer A, Puddey IB. Admission selection criteria as predictors of outcomes in an undergraduate medical course: a prospective study. Med Teach 2011;33:997-1004. https://doi.org/10.3109/0142159X.2011.577123 |
| 5 | Shulruf B, Poole P, Wang GY, Rudland J, Wilkinson T. How well do selection tools predict performance later in a medical programme? Adv Health Sci Educ Theory Pract 2012;17:615-626. https://doi.org/10.1007/s10459-011-9324-1 |

*4) End of program academic results: 2 effect sizes, 2 articles*

| No. | Article |
| --- | --- |
| 1 | Mercer A, Puddey IB. Admission selection criteria as predictors of outcomes in an undergraduate medical course: a prospective study. Med Teach 2011;33:997-1004. https://doi.org/10.3109/0142159X.2011.577123 |
| 2 | Wilkinson D, Zhang J, Parker M. Predictive validity of the Undergraduate Medicine and Health Sciences Admission Test for medical students’ academic performance. Med J Aust 2011;194:341-344. https://doi.org/10.5694/j.1326-5377.2011.tb03002.x |

*5) Dropout: nil*

***d. Verbal reasoning***

*1) Early OSCE: nil*

*2) End of program OSCE: 4 effect sizes, 4 articles*

| No. | Article |
| --- | --- |
| 1 | Adam J, Bore M, McKendree J, Munro D, Powis D. Can personal qualities of medical students predict in-course examination success and professional behaviour?: an exploratory prospective cohort study. BMC Med Educ 2012;12:69. https://doi.org/10.1186/1472-6920-12-69 |
| 2 | Adam J, Bore M, Childs R, Dunn J, Mckendree J, Munro D, Powis D. Predictors of professional behaviour and academic outcomes in a UK medical school: a longitudinal cohort study. Med Teach 2015;37:868-880. https://doi.org/10.3109/0142159X.2015.1009023 |
| 3 | McManus IC, Dewberry C, Nicholson S, Dowell JS. The UKCAT-12 study: educational attainment, aptitude test performance, demographic and socio-economic contextual factors as predictors of first year outcome in a cross-sectional collaborative study of 12 UK medical schools. BMC Med 2013;11:244. https://doi.org/10.1186/1741-7015-11-244 |
| 4 | Yates J, James D. The value of the UK Clinical Aptitude Test in predicting pre-clinical performance: a prospective cohort study at Nottingham Medical School. BMC Med Educ 2010;10:55. https://doi.org/10.1186/1472-6920-10-55 |

*3) Early academic results: 4 effect sizes, 3 articles*

| No. | Article |
| --- | --- |
| 1 | Yates J, James D. The value of the UK Clinical Aptitude Test in predicting pre-clinical performance: a prospective cohort study at Nottingham Medical School. BMC Med Educ 2010;10:55. https://doi.org/10.1186/1472-6920-10-55 |
| 2 | Cerutti B, Bernheim L, van Gessel E. The predictive validity of the aptitude test for the performance of students starting a medical curriculum. Swiss Med Wkly 2013;143:w13872. https://doi.org/10.4414/smw.2013.13872 |
| 3 | Adam J, Bore M, McKendree J, Munro D, Powis D. Can personal qualities of medical students predict in-course examination success and professional behaviour?: an exploratory prospective cohort study. BMC Med Educ 2012;12:69. https://doi.org/10.1186/1472-6920-12-69 |

*4) End of program academic results: 3 effect sizes, 3 articles*

| No. | Article |
| --- | --- |
| 1 | MacKenzie RK, Cleland JA, Ayansina D, Nicholson S. Does the UKCAT predict performance on exit from medical school?: a national cohort study. BMJ Open 2016;6:e011313. https://doi.org/10.1136/bmjopen-2016-011313 |
| 2 | McManus IC, Dewberry C, Nicholson S, Dowell JS. The UKCAT-12 study: educational attainment, aptitude test performance, demographic and socio-economic contextual factors as predictors of first year outcome in a cross-sectional collaborative study of 12 UK medical schools. BMC Med 2013;11:244. https://doi.org/10.1186/1741-7015-11-244 |
| 3 | Yates J, James D. The UK Clinical Aptitude Test and clinical course performance at Nottingham: a prospective cohort study. BMC Med Educ 2013;13:32. https://doi.org/10.1186/1472-6920-13-32 |

*5) Dropout: nil*

***e. Quantitative reasoning***

*1) Early OSCE: nil*

*2) End of program OSCE: 3 effect sizes, 3 articles*

| No. | Article |
| --- | --- |
| 1 | Adam J, Bore M, McKendree J, Munro D, Powis D. Can personal qualities of medical students predict in-course examination success and professional behaviour?: an exploratory prospective cohort study. BMC Med Educ 2012;12:69. https://doi.org/10.1186/1472-6920-12-69 |
| 2 | McManus IC, Dewberry C, Nicholson S, Dowell JS. The UKCAT-12 study: educational attainment, aptitude test performance, demographic and socio-economic contextual factors as predictors of first year outcome in a cross-sectional collaborative study of 12 UK medical schools. BMC Med 2013;11:244. https://doi.org/10.1186/1741-7015-11-244 |
| 3 | Yates J, James D. The value of the UK Clinical Aptitude Test in predicting pre-clinical performance: a prospective cohort study at Nottingham Medical School. BMC Med Educ 2010;10:55. https://doi.org/10.1186/1472-6920-10-55 |

*3) Early academic results: 4 effect sizes, 3 articles*

| No. | Article |
| --- | --- |
| 1 | Adam J, Bore M, McKendree J, Munro D, Powis D. Can personal qualities of medical students predict in-course examination success and professional behaviour?: an exploratory prospective cohort study. BMC Med Educ 2012;12:69. https://doi.org/10.1186/1472-6920-12-69 |
| 2 | Cerutti B, Bernheim L, van Gessel E. The predictive validity of the aptitude test for the performance of students starting a medical curriculum. Swiss Med Wkly 2013;143:w13872. https://doi.org/10.4414/smw.2013.13872 |
| 3 | Yates J, James D. The value of the UK Clinical Aptitude Test in predicting pre-clinical performance: a prospective cohort study at Nottingham Medical School. BMC Med Educ 2010;10:55. https://doi.org/10.1186/1472-6920-10-55 |

*4) End of program academic results: 3 effect sizes, 3 articles*

| No. | Article |
| --- | --- |
| 1 | MacKenzie RK, Cleland JA, Ayansina D, Nicholson S. Does the UKCAT predict performance on exit from medical school?: a national cohort study. BMJ Open 2016;6:e011313. https://doi.org/10.1136/bmjopen-2016-011313 |
| 2 | McManus IC, Dewberry C, Nicholson S, Dowell JS. The UKCAT-12 study: educational attainment, aptitude test performance, demographic and socio-economic contextual factors as predictors of first year outcome in a cross-sectional collaborative study of 12 UK medical schools. BMC Med 2013;11:244. https://doi.org/10.1186/1741-7015-11-244 |
| 3 | Yates J, James D. The UK Clinical Aptitude Test and clinical course performance at Nottingham: a prospective cohort study. BMC Med Educ 2013;13:32. https://doi.org/10.1186/1472-6920-13-32 |

*5) Dropout: nil*

**3. Interviews**

***a. Multiple mini-interview (MMI)***

*1) Early OSCE: 6 effect sizes, 5 articles*

| No. | Title |
| --- | --- |
| 1 | Grone O, Mielke I, Knorr M, Ehrhardt M, Bergelt C. Associations between communication OSCE performance and admission interviews in medical education. Patient Educ Couns 2022;105:2270-2275. https://doi.org/10.1016/j.pec.2021.11.005 |
| 2 | Husbands A, Dowell J. Predictive validity of the Dundee multiple mini-interview. Med Educ 2013;47:717-725. https://doi.org/10.1111/medu.12193 |
| 3 | Knorr M, Schwibbe A, Ehrhardt M, Lackamp J, Zimmermann S, Hampe W. Validity evidence for the Hamburg multiple mini-interview. BMC Med Educ 2018;18:106. https://doi.org/10.1186/s12909-018-1208-0 |
| 4 | Knorr M, Meyer H, Sehner S, Hampe W, Zimmermann S. Exploring sociodemographic subgroup differences in multiple mini-interview (MMI) performance based on MMI station type and the implications for the predictive fairness of the Hamburg MMI. BMC Med Educ 2019;19:243. https://doi.org/10.1186/s12909-019-1674-z |
| 5 | Rauf A, Tayyab A, Masrur A. Relationship between student performances in non-cognitive skills in multiple mini interview and integrated practical examination. J Coll Physicians Surg Pak 2018;28:270-273. https://doi.org/10.29271/jcpsp.2018.04.270 |

*2) End of program OSCE: nil*

*3) Early academic: 4 effect sizes, 3 articles*

| No. | Article |
| --- | --- |
| 1 | Griffin B, Bayl-Smith P, Hu W. Predicting patterns of change and stability in student performance across a medical degree. Med Educ 2018;52:438-446. https://doi.org/10.1111/medu.13508 |
| 2 | Husbands A, Dowell J. Predictive validity of the Dundee multiple mini-interview. Med Educ 2013;47:717-725. https://doi.org/10.1111/medu.12193 |
| 3 | Irasanti SN, Akbar IB, Dewi MK, Susanti Y. The capability of selection tools to predict future academic performance of medical students. J Phys Conf Ser 2020;1469:012138. https://doi.org/10.1088/1742-6596/1469/1/012138 |

*4) End of program academic: 1 effect size, 1 article*

| No. | Article |
| --- | --- |
| 1 | Eva KW, Reiter HI, Rosenfeld J, Trinh K, Wood TJ, Norman GR. Association between a medical school admission process using the multiple mini-interview and national licensing examination scores. JAMA 2012;308:2233-2240. https://doi.org/10.1001/jama.2012.36914 |

*5) Dropout: 1 effect size, 1 article*

| No. | Article |
| --- | --- |
| 1 | Hefny AF, Almansoori TM, El-Zubeir M, AlBawardi A, Shaban S, Magzoub ME, Zoubeidi T, Mansour NA. Relationship between admission selection tools and student attrition in the early years of medical school. J Taibah Univ Med Sci 2024;19:447-452. https://doi.org/10.1016/j.jtumed.2024.02.004 |

***b. Panel***

*1) Early OSCE: 3 effect sizes, 3 articles*

| No. | Article |
| --- | --- |
| 1 | Casey M, Wilkinson D, Fitzgerald J, Eley D, Connor J. Clinical communication skills learning outcomes among first year medical students are consistent irrespective of participation in an interview for admission to medical school. Med Teach 2014;36:640-642. https://doi.org/10.3109/0142159X.2014.907880 |
| 2 | Simpson PL, Scicluna HA, Jones PD, Cole AM, O’Sullivan AJ, Harris PG, Velan G, McNeil HP. Predictive validity of a new integrated selection process for medical school admission. BMC Med Educ 2014;14:86. https://doi.org/10.1186/1472-6920-14-86 |
| 3 | Tsikas SA. Can selection interviews predict OSCE performance?: evidence from Hannover Medical School. Z Evid Fortbild Qual Gesundhwes 2022;173:85-91. https://doi.org/10.1016/j.zefq.2022.05.008 |

*2) End of program OSCE: 5 effect sizes, 4 articles*

| No. | Article |
| --- | --- |
| 1 | Dahlin M, Soderberg S, Holm U, Nilsson I, Farnebo LO. Comparison of communication skills between medical students admitted after interviews or on academic merits. BMC Med Educ 2012;12:46. https://doi.org/10.1186/1472-6920-12-46 |
| 2 | Husbands A, Mathieson A, Dowell J, Cleland J, MacKenzie R. Predictive validity of the UK clinical aptitude test in the final years of medical school: a prospective cohort study. BMC Med Educ 2014;14:88. https://doi.org/10.1186/1472-6920-14-88. |
| 3 | Simpson PL, Scicluna HA, Jones PD, Cole AM, O’Sullivan AJ, Harris PG, Velan G, McNeil HP. Predictive validity of a new integrated selection process for medical school admission. BMC Med Educ 2014;14:86. https://doi.org/10.1186/1472-6920-14-86 |
| 4 | Yusoff MS. The outcomes that an interview-based medical school admission process has on academic performance, psychological health, personality traits, and emotional intelligence. J Taibah Univ Med Sci 2018;13:503-511. https://doi.org/10.1016/j.jtumed.2018.09.003 |

*3) Early academic results: 6 effect sizes, 6 articles*

| No. | Article |
| --- | --- |
| 1 | Alnasir FA, Jaradat AA. The effectiveness of AGU-MCAT in predicting medical student performance in year one of the College of Medicine of the Arabian Gulf University. Educ Health (Abingdon) 2011;24:447. https://doi.org/10.4103/1357-6283.101444 |
| 2 | Edwards D, Friedman T, Pearce J. Same admissions tools, different outcomes: a critical perspective on predictive validity in three undergraduate medical schools. BMC Med Educ 2013;13:173. https://doi.org/10.1186/1472-6920-13-173 |
| 3 | Fan AP, Tsai TC, Su TP, Kosik RO, Morisky DE, Chen CH, Shih WJ, Lee CH. A longitudinal study of the impact of interviews on medical school admissions in Taiwan. Eval Health Prof 2010;33:140-163. https://doi.org/10.1177/0163278710361920 |
| 4 | Mercer A, Puddey IB. Admission selection criteria as predictors of outcomes in an undergraduate medical course: a prospective study. Med Teach 2011;33:997-1004. https://doi.org/10.3109/0142159X.2011.577123 |
| 5 | Shulruf B, Poole P, Wang GY, Rudland J, Wilkinson T. How well do selection tools predict performance later in a medical programme? Adv Health Sci Educ Theory Pract 2012;17:615-626. https://doi.org/10.1007/s10459-011-9324-1 |
| 6 | Simpson PL, Scicluna HA, Jones PD, Cole AM, O’Sullivan AJ, Harris PG, Velan G, McNeil HP. Predictive validity of a new integrated selection process for medical school admission. BMC Med Educ 2014;14:86. https://doi.org/10.1186/1472-6920-14-86 |

*4) End of program academic results: 5 effect sizes, 5 articles*

| No. | Article |
| --- | --- |
| 1 | Al-Rukban MO, Munshi FM, Abdulghani HM, Al-Hoqail I. The ability of the pre-admission criteria to predict performance in a Saudi medical school. Saudi Med J 2010;31:560-564. |
| 2 | Fan AP, Tsai TC, Su TP, Kosik RO, Morisky DE, Chen CH, Shih WJ, Lee CH. A longitudinal study of the impact of interviews on medical school admissions in Taiwan. Eval Health Prof 2010;33:140-163. https://doi.org/10.1177/0163278710361920 |
| 3 | Mercer A, Puddey IB. Admission selection criteria as predictors of outcomes in an undergraduate medical course: a prospective study. Med Teach 2011;33:997-1004. https://doi.org/10.3109/0142159X.2011.577123 |
| 4 | Simpson PL, Scicluna HA, Jones PD, Cole AM, O’Sullivan AJ, Harris PG, Velan G, McNeil HP. Predictive validity of a new integrated selection process for medical school admission. BMC Med Educ 2014;14:86. https://doi.org/10.1186/1472-6920-14-86 |
| 5 | Yusoff MS. The outcomes that an interview-based medical school admission process has on academic performance, psychological health, personality traits, and emotional intelligence. J Taibah Univ Med Sci 2018;13:503-511. https://doi.org/10.1016/j.jtumed.2018.09.003 |

*5) Dropout: 1 effect size, 1 article*

| No. | Title |
| --- | --- |
| 1 | Shulruf B, Poole P, Wang GY, Rudland J, Wilkinson T. How well do selection tools predict performance later in a medical programme? Adv Health Sci Educ Theory Pract 2012;17:615-626. https://doi.org/10.1007/s10459-011-9324-1 |

**4. Situational judgement test**

***a. Early OSCE: nil***

***b. End of program OSCE: nil***

***c. Early academic results: 8 effect size, 2 article***

| No. | Article |
| --- | --- |
| 1 | Lievens F. Adjusting medical school admission: assessing interpersonal skills using situational judgement tests. Med Educ 2013;47:182-189. https://doi.org/10.1111/medu.12089 |
| 2 | Ballejos MP, Cestone C, Copeland HL, Dunleavy DM, Geiger T, Patel D. Predicting medical student performance with a situational judgment test in admissions. Acad Med 2024;99:175-182. https://doi.org/10.1097/ACM.0000000000005516 |

***d. End of program academic results: 2 effect sizes, 2 articles***

| No. | Article |
| --- | --- |
| 1 | Lievens F. Adjusting medical school admission: assessing interpersonal skills using situational judgement tests. Med Educ 2013;47:182-189. https://doi.org/10.1111/medu.12089 |
| 2 | Lievens F, Sackett PR. The validity of interpersonal skills assessment via situational judgment tests for predicting academic success and job performance. J Appl Psychol 2012;97:460-468. https://doi.org/10.1037/a0025741 |

***e. Dropout: nil***

**5. Personality testing**

***a. Personal Qualities Assessment (PQA)***

*1) Early OSCE: 1 effect size, 1 article*

| No. | Article |
| --- | --- |
| 1 | Dowell J, Lumsden MA, Powis D, Munro D, Bore M, Makubate B, Kumwenda B. Predictive validity of the personal qualities assessment for selection of medical students in Scotland. Med Teach 2011;33:e485-e488. https://doi.org/10.3109/0142159X.2011.599448 |

*2) End of program OSCE: 1 effect size, 1 article*

| No. | Article |
| --- | --- |
| 1 | Adam J, Bore M, Childs R, Dunn J, Mckendree J, Munro D, Powis D. Predictors of professional behaviour and academic outcomes in a UK medical school: a longitudinal cohort study. Med Teach 2015;37:868-880. https://doi.org/10.3109/0142159X.2015.1009023 |

*2) Early academic results: nil*

*3) End of program academic results: nil*

*4) Dropout: nil*

***b. Minnesota Multiphasic Personality Inventory***

*1) Early OSCE: nil*

*2) End of program OSCE: nil*

*3) Early academic results: 1 effect size, 1 article*

| No. | Article |
| --- | --- |
| 1 | Irasanti SN, Akbar IB, Dewi MK, Susanti Y. The capability of selection tools to predict future academic performance of medical students. J Phys Conf Ser 2020;1469:012138. https://doi.org/10.1088/1742-6596/1469/1/012138 |

*4) End of program academic results: nil*

*5) Dropout: nil*
