# Supplementary material for: Insights into undergraduate medical student selection tools: a systematic review and meta-analysis
Source: J Educ Eval Health Prof. 2024 Dec 12;21:41. doi: 10.3352/jeehp.2024.21.22 (PMC11494217; doi:10.3352/jeehp.2024.21.22)
Supplement: Supplementary file 5 — Supplement 4. Bias appraisal. [file jeehp-21-22-suppl4.docx]

**Supplement 4.** Bias appraisal

| Article | 1. Is the source population representative of the population of interest? | 2. Is the response rate adequate? | 3. Is there little missing data?^a)^ | 4. Is the survey clinically sensible? | 5. Is there any evidence for the reliability and validity of the survey instrument? |
| --- | --- | --- | --- | --- | --- |
| Abbiati M, Cerutti B. Do students’ personality traits change during medical training?: a longitudinal cohort study. Adv Health Sci Educ Theory Pract 2023;28:1079-1092. https://doi.org/10.1007/s10459-023-10205-2 | Probably yes | NA^b)^ | Probably yes | NA^c)^ | NA^d)^ |
| Adam J, Bore M, McKendree J, Munro D, Powis D. Can personal qualities of medical students predict in-course examination success and professional behaviour?: an exploratory prospective cohort study. BMC Med Educ 2012;12:69. https://doi.org/10.1186/1472-6920-12-69 | Probably yes | NA^b)^ | Definitely yes (low risk of bias) | NA^c)^ | NA^d)^ |
| Adam J, Bore M, Childs R, Dunn J, Mckendree J, Munro D, Powis D. Predictors of professional behaviour and academic outcomes in a UK medical school: a longitudinal cohort study. Med Teach 2015;37:868-880. https://doi.org/10.3109/0142159X.2015.1009023 | Probably yes | NA^b)^ | Probably yes | NA^c)^ | NA^d)^ |
| Adeniyi OS, Araoye MA, Amali EO, Eru EU, Ojabo CO, Alao OO. Effect of using combination of O’level result with JAMB score on student performance in the first two years of medical school in Benue State University, Makurdi. Afr J Biomed Res 2010;13:189-195. | Probably yes | NA^b)^ | Definitely yes (low risk of bias) | NA^c)^ | NA^d)^ |
| Al Alwan I, Al Kushi M, Tamim H, Magzoub M, Elzubeir M. Health sciences and medical college preadmission criteria and prediction of in-course academic performance: a longitudinal cohort study. Adv Health Sci Educ Theory Pract 2013;18:427-438. https://doi.org/10.1007/s10459-012-9380-1 | Probably yes | NA^b)^ | Definitely yes (low risk of bias) | NA^c)^ | NA^d)^ |
| Albishri JA, Aly SM, Alnemary Y. Admission criteria to Saudi medical schools: which is the best predictor for successful achievement? Saudi Med J 2012;33:1222-1226. | Probably yes | NA^b)^ | Definitely yes (low risk of bias) | NA^c)^ | NA^d)^ |
| Alhadlaq AM, Alshammari OF, Alsager SM, Neel KA, Mohamed AG. Ability of admissions criteria to predict early academic performance among students of health science colleges at King Saud University, Saudi Arabia. J Dent Educ. 2015 Jun;79(6):665-70. https://doi.org/10.1002/j.0022-0337.2015.79.6.tb05939.x | Probably yes | NA^b)^ | Definitely yes (low risk of bias) | NA^c)^ | NA^d)^ |
| Almarabheh A, Shehata MH, Ismaeel A, Atwa H, Jaradat A. Predictive validity of admission criteria in predicting academic performance of medical students: a retrospective cohort study. Front Med (Lausanne) 2022;9:971926. https://doi.org/10.3389/fmed.2022.971926 | Probably yes | NA^b)^ | Definitely yes (low risk of bias) | NA^c)^ | NA^d)^ |
| Alnasir FA, Jaradat AA. The effectiveness of AGU-MCAT in predicting medical student performance in year one of the College of Medicine of the Arabian Gulf University. Educ Health (Abingdon) 2011;24:447. https://doi.org/10.4103/1357-6283.101444 | Probably yes | NA^b)^ | Definitely yes (low risk of bias) | NA^c)^ | NA^d)^ |
| Al-Rukban MO, Munshi FM, Abdulghani HM, Al-Hoqail I. The ability of the pre-admission criteria to predict performance in a Saudi medical school. Saudi Med J 2010;31:560-564. | Probably yes | NA^b)^ | Definitely yes (low risk of bias) | NA^c)^ | NA^d)^ |
| Althewini A, Al Baz N. Prediction of Admission Tests for medical students’ academic performance. Adv Med Educ Pract 2022;13:1287-1292. https://doi.org/10.2147/AMEP.S355474 | Probably yes | NA^b)^ | Definitely yes (low risk of bias) | NA^c)^ | NA^d)^ |
| Amelung D, Zegota S, Espe L, Wittenberg T, Raupach T, Kadmon M. Considering vocational training as selection criterion for medical students: evidence for predictive validity. Adv Health Sci Educ Theory Pract 2022;27:933-948. https://doi.org/10.1007/s10459-022-10120-y | Probably yes | NA^b)^ | Definitely no (high risk of bias) | NA^c)^ | NA^d)^ |

| Article | 1. Is the source population representative of the population of interest? | 2. Is the response rate adequate? | 3. Is there little missing data?^a)^ | 4. Is the survey clinically sensible? | 5. Is there any evidence for the reliability and validity of the survey instrument? |
| --- | --- | --- | --- | --- | --- |
| Ballejos MP, Cestone C, Copeland HL, Dunleavy DM, Geiger T, Patel D. Predicting medical student performance with a situational judgment test in admissions. Acad Med 2024;99:175-182. https://doi.org/10.1097/ACM.0000000000005516 | Probably yes | NA^b)^ | Definitely yes (low risk of bias) | NA^c)^ | NA^d)^ |
| Bekele AT, Beza SW, Gedamu S, Berndt M. Predictors of college academic achievement for medical students: the case of Gondar University, College of Medicine and Health Sciences, Ethiopia. Adv Med Educ Pract 2023;14:603-613. https://doi.org/10.2147/AMEP.S406031 | Probably yes | NA^b)^ | Definitely yes (low risk of bias) | NA^c)^ | NA^d)^ |
| Bestetti RB, Durand MT, Couto LB, Faria-Jr M, Fumagalli HF, Silva VMR, Romão GS, Furlan-Daniel R, Garcia ME, Ferri SMN, Reis ACS, Jorge-Neto SD, Geleilete TJM. A Comparison of the Academic Achievement at the End of the Medicine Undergraduate Degree Program Between Students Who Only Used the University Admission Test and Those Who Used the University Admission Test Plus Marks from the High School National Exam (ENEM) at a Single Brazilian Center. Adv Med Educ Pract 2023;14:1185-1190. https://doi.org/10.2147/AMEP.S372822 | Probably yes | NA^b)^ | Probably yes | NA^c)^ | NA^d)^ |
| Bestetti RB, Couto LB, Roncato-Paiva P, Romão GS, Faria-Jr M, Furlan-Daniel RA, Geleilete TJM, Jorge-Neto SD, Mendonça FP, Garcia ME, Durand MT. University admission test associates with academic performance at the end of medical course in a PBL medical hybrid curriculum. Adv Med Educ Pract 2020;11:579-585. https://doi.org/10.2147/AMEP.S255732 | Probably yes | NA^b)^ | Definitely yes (low risk of bias) | NA^c)^ | NA^d)^ |
| Bußenius L, Harendza S. Are different medical school admission tests associated with the outcomes of a simulation-based OSCE? BMC Med Educ 2021;21:263. https://doi.org/10.1186/s12909-021-02703-x | Probably yes | NA^b)^ | Definitely no (high risk of bias) | NA^c)^ | NA^d)^ |
| Casey M, Wilkinson D, Fitzgerald J, Eley D, Connor J. Clinical communication skills learning outcomes among first year medical students are consistent irrespective of participation in an interview for admission to medical school. Med Teach 2014;36:640-642. https://doi.org/10.3109/0142159X.2014.907880 | Probably yes | NA^b)^ | Definitely yes (low risk of bias) | NA^c)^ | NA^d)^ |
| Cerutti B, Bernheim L, van Gessel E. The predictive validity of the aptitude test for the performance of students starting a medical curriculum. Swiss Med Wkly 2013;143:w13872. https://doi.org/10.4414/smw.2013.13872 | Definitely yes (low risk of bias) | NA^b)^ | Definitely yes (low risk of bias) | NA^c)^ | NA^d)^ |
| Dahlin M, Söderberg S, Holm U, Nilsson I, Farnebo LO. Comparison of communication skills between medical students admitted after interviews or on academic merits. BMC Med Educ 2012;12:46. https://doi.org/10.1186/1472-6920-12-46 | Definitely yes (low risk of bias) | NA^b)^ | Definitely yes (low risk of bias) | NA^c)^ | NA^d)^ |
| Davies DJ, Sam AH, Murphy KG, Khan SA, Choe R, Cleland J. BMAT's predictive validity for medical school performance: a retrospective cohort study. Med Educ 2022;56:936-948. https://doi.org/10.1111/medu.14819 | Probably yes | NA^b)^ | Probably yes | NA^c)^ | NA^d)^ |
| Dowell J, Lumsden MA, Powis D, Munro D, Bore M, Makubate B, Kumwenda B. Predictive validity of the personal qualities assessment for selection of medical students in Scotland. Med Teach 2011;33:e485-e488. https://doi.org/10.3109/0142159X.2011.599448 | Definitely yes (low risk of bias) | NA^b)^ | Probably yes | NA^c)^ | NA^d)^ |
| Edwards D, Friedman T, Pearce J. Same admissions tools, different outcomes: a critical perspective on predictive validity in three undergraduate medical schools. BMC Med Educ 2013;13:173. https://doi.org/10.1186/1472-6920-13-173 | Probably yes | NA^b)^ | Probably yes | NA^c)^ | NA^d)^ |
| Eva KW, Reiter HI, Rosenfeld J, Trinh K, Wood TJ, Norman GR. Association between a medical school admission process using the multiple mini-interview and national licensing examination scores. JAMA 2012;308:2233-2240. https://doi.org/10.1001/jama.2012.36914 | Probably yes | NA^b)^ | Probably yes | NA^c)^ | NA^d)^ |

| Article | 1. Is the source population representative of the population of interest? | 2. Is the response rate adequate? | 3. Is there little missing data?^a)^ | 4. Is the survey clinically sensible? | 5. Is there any evidence for the reliability and validity of the survey instrument? |
| --- | --- | --- | --- | --- | --- |
| Fan AP, Tsai TC, Su TP, Kosik RO, Morisky DE, Chen CH, Shih WJ, Lee CH. A longitudinal study of the impact of interviews on medical school admissions in Taiwan. Eval Health Prof 2010;33:140-163. https://doi.org/10.1177/0163278710361920 | Probably yes | NA^b)^ | Probably yes | NA^c)^ | NA^d)^ |
| Gautam AP, Paudel BH, Agrawal CS, Niraula SR, Dalen JV. Examination of relationship of scores obtained in grades 10 and 12 with the entry and success in undergraduate medical education. Kathmandu Univ Med J (KUMJ) 2012;10:66-71. https://doi.org/10.3126/kumj.v10i1.6918 | Probably yes | NA^b)^ | Definitely yes (low risk of bias) | NA^c)^ | NA^d)^ |
| Gebru HT, Verstegen D. Assessing predictors of students’ academic performance in Ethiopian new medical schools: a concurrent mixed-method study. BMC Med Educ 2023;23:448. https://doi.org/10.1186/s12909-023-04372-4 | Definitely yes (low risk of bias) | NA^b)^ | Probably yes | NA^c)^ | NA^d)^ |
| Griffin B, Bayl-Smith P, Hu W. Predicting patterns of change and stability in student performance across a medical degree. Med Educ 2018;52:438-446. https://doi.org/10.1111/medu.13508 | Probably yes | NA^b)^ | Probably yes | NA^c)^ | NA^d)^ |
| Gröne O, Mielke I, Knorr M, Ehrhardt M, Bergelt C. Associations between communication OSCE performance and admission interviews in medical education. Patient Educ Couns 2022;105:2270-2275. https://doi.org/10.1016/j.pec.2021.11.005 | Probably yes | NA^b)^ | Probably yes | NA^c)^ | NA^d)^ |
| Guraya SY. High school grades are not reliable predictors of academic performance in undergraduate medical school: a study from a Saudi medical school. Biomed Pharmacol J 2015;5:219-225. http://biomedpharmajournal.org/?p=2475 | Probably yes | NA^b)^ | Probably yes | NA^c)^ | NA^d)^ |
| Hefny AF, Almansoori TM, El-Zubeir M, AlBawardi A, Shaban S, Magzoub ME, Zoubeidi T, Mansour NA. Relationship between admission selection tools and student attrition in the early years of medical school. J Taibah Univ Med Sci 2024;19:447-452. https://doi.org/10.1016/j.jtumed.2024.02.004 | Probably yes | NA^b)^ | Probably yes | NA^c)^ | NA^d)^ |
| Hendi A, Mahfouz MS, Alqassim AY, Makeen A, Somaili M, Shami MO, Names AA, Darraj A, Kariri A, Ashiri A, Alhazmi AH. Admission grades as predictors of medical students’ academic performance: a cross-sectional study from Saudi Arabia. Eur J Investig Health Psychol Educ 2022;12:1572-1580. https://doi.org/10.3390/ejihpe12110110 | Probably yes | NA^b)^ | Probably yes | NA^c)^ | NA^d)^ |
| Hewage SN, Salgado LS, Fernando GM, Liyanage PL, Pathmeswaran A, de Silva NR. Selection of medical students in Sri Lanka: time to re-think criteria? Ceylon Med J 2011;56:22-28. https://doi.org/10.4038/cmj.v56i1.2891 | Probably yes | NA^b)^ | Probably yes | NA^c)^ | NA^d)^ |
| Husbands A, Dowell J. Predictive validity of the Dundee multiple mini-interview. Med Educ 2013;47:717-725. https://doi.org/10.1111/medu.12193 | Probably yes | NA^b)^ | Probably yes | NA^c)^ | NA^d)^ |
| Husbands A, Mathieson A, Dowell J, Cleland J, MacKenzie R. Predictive validity of the UK clinical aptitude test in the final years of medical school: a prospective cohort study. BMC Med Educ 2014;14:88. https://doi.org/10.1186/1472-6920-14-88. | Probably yes | NA^b)^ | Probably yes | NA^c)^ | NA^d)^ |
| Irasanti SN, Akbar IB, Dewi MK, Susanti Y. The capability of selection tools to predict future academic performance of medical students. J Phys Conf Ser 2020;1469:012138. https://doi.org/10.1088/1742-6596/1469/1/012138 | Probably yes | NA^b)^ | Probably yes | NA^c)^ | NA^d)^ |
| Kadmon G, Kadmon M. Academic performance of students with the highest and mediocre school-leaving grades: does the aptitude test for medical studies (TMS) balance their prognoses? GMS J Med Educ 2016;33:Doc7. https://doi.org/10.3205/zma001006 | Probably yes | NA^b)^ | Probably yes | NA^c)^ | NA^d)^ |

| Article | 1. Is the source population representative of the population of interest? | 2. Is the response rate adequate? | 3. Is there little missing data?^a)^ | 4. Is the survey clinically sensible? | 5. Is there any evidence for the reliability and validity of the survey instrument? |
| --- | --- | --- | --- | --- | --- |
| Kelly ME, Regan D, Dunne F, Henn P, Newell J, O'Flynn S. To what extent does the Health Professions Admission Test-Ireland predict performance in early undergraduate tests of communication and clinical skills?: an observational cohort study. BMC Med Educ 2013;13:68. https://doi.org/10.1186/1472-6920-13-68 | Probably yes | NA^b)^ | Probably yes | NA^c)^ | NA^d)^ |
| Knorr M, Schwibbe A, Ehrhardt M, Lackamp J, Zimmermann S, Hampe W. Validity evidence for the Hamburg multiple mini-interview. BMC Med Educ 2018;18:106. https://doi.org/10.1186/s12909-018-1208-0 | Probably yes | NA^b)^ | Probably No | NA^c)^ | NA^d)^ |
| Knorr M, Meyer H, Sehner S, Hampe W, Zimmermann S. Exploring sociodemographic subgroup differences in multiple mini-interview (MMI) performance based on MMI station type and the implications for the predictive fairness of the Hamburg MMI. BMC Med Educ 2019;19:243. https://doi.org/10.1186/s12909-019-1674-z | Probably yes | NA^b)^ | Probably yes | NA^c)^ | NA^d)^ |
| Kraft HG, Lamina C, Kluckner T, Wild C, Prodinger WM. Paradise lost or paradise regained?: changes in admission system affect academic performance and drop-out rates of medical students. Med Teach 2013;35:e1123-e1129. https://doi.org/10.3109/0142159X.2012.733835 | Definitely yes (low risk of bias) | NA^b)^ | Probably yes | NA^c)^ | NA^d)^ |
| Krings R, Huwendiek S, Walsh N, Stricker D, Berendonk C. Predictive power of high school educational attainment and the medical aptitude test for performance during the Bachelor program in human medicine at the University of Bern: a cohort study. Swiss Med Wkly 2020;150:w20389. https://doi.org/10.4414/smw.2020.20389 | Probably yes | NA^b)^ | Probably yes | NA^c)^ | NA^d)^ |
| Maslov Kruzicevic S, Barisic KJ, Banozic A, Esteban CD, Sapunar D, Puljak L. Predictors of attrition and academic success of medical students: a 30-year retrospective study. PLoS One 2012;7:e39144. https://doi.org/10.1371/journal.pone.0039144 | Probably yes | NA^b)^ | Probably yes | NA^c)^ | NA^d)^ |
| Lievens F. Adjusting medical school admission: assessing interpersonal skills using situational judgement tests. Med Educ 2013;47:182-189. https://doi.org/10.1111/medu.12089 | Definitely yes (low risk of bias) | NA^b)^ | Probably yes | NA^c)^ | NA^d)^ |
| Lievens F, Sackett PR. The validity of interpersonal skills assessment via situational judgment tests for predicting academic success and job performance. J Appl Psychol 2012;97:460-468. https://doi.org/10.1037/a0025741 | Definitely yes (low risk of bias) | NA^b)^ | Probably yes | NA^c)^ | NA^d)^ |
| MacKenzie RK, Cleland JA, Ayansina D, Nicholson S. Does the UKCAT predict performance on exit from medical school?: a national cohort study. BMJ Open 2016;6:e011313. https://doi.org/10.1136/bmjopen-2016-011313 | Definitely yes (low risk of bias) | NA^b)^ | Probably yes | NA^c)^ | NA^d)^ |
| McManus IC, Dewberry C, Nicholson S, Dowell JS. The UKCAT-12 study: educational attainment, aptitude test performance, demographic and socio-economic contextual factors as predictors of first year outcome in a cross-sectional collaborative study of 12 UK medical schools. BMC Med 2013;11:244. https://doi.org/10.1186/1741-7015-11-244 | Definitely yes (low risk of bias) | NA^b)^ | Probably yes | NA^c)^ | NA^d)^ |
| McManus IC, Woolf K, Harrison D, Tiffin PA, Paton LW, Cheung KYF, Smith DT. Predictive validity of A-level grades and teacher-predicted grades in UK medical school applicants: a retrospective analysis of administrative data in a time of COVID-19. BMJ Open 2021;11:e047354. https://doi.org/10.1136/bmjopen-2020-047354 | Definitely yes (low risk of bias) | NA^b)^ | Probably yes | NA^c)^ | NA^d)^ |
| McManus IC, Woolf K, Dacre J, Paice E, Dewberry C. The Academic Backbone: longitudinal continuities in educational achievement from secondary school and medical school to MRCP(UK) and the specialist register in UK medical students and doctors. BMC Med 2013;11:242. https://doi.org/10.1186/1741-7015-11-242 | Probably yes | NA^b)^ | Probably yes | NA^c)^ | NA^d)^ |
| Mercer A, Puddey IB. Admission selection criteria as predictors of outcomes in an undergraduate medical course: a prospective study. Med Teach 2011;33:997-1004. https://doi.org/10.3109/0142159X.2011.577123 | Probably yes | NA^b)^ | Probably yes | NA^c)^ | NA^d)^ |

| Article | 1. Is the source population representative of the population of interest? | 2. Is the response rate adequate? | 3. Is there little missing data?^a)^ | 4. Is the survey clinically sensible? | 5. Is there any evidence for the reliability and validity of the survey instrument? |
| --- | --- | --- | --- | --- | --- |
| Meyer H, Zimmermann S, Hissbach J, Klusmann D, Hampe W. Selection and academic success of medical students in Hamburg, Germany. BMC Med Educ 2019;19:23. https://doi.org/10.1186/s12909-018-1443-4 | Probably yes | NA^b)^ | Probably yes | NA^c)^ | NA^d)^ |
| Mirgani Z, Shantakumari N, Hassan I. Predictors of student performance in foundation year of medical school. Sys Rev Pharm 2020;11:201-205. | Probably yes | NA^b)^ | Definitely yes (low risk of bias) | NA^c)^ | NA^d)^ |
| Ozeki S, Kasamo S, Inoue H, Matsumoto S. Does regional quota status affect the performance of undergraduate medical students in Japan?: a 10-year analysis. Int J Med Educ 2022;13:307-314. https://doi.org/10.5116/ijme.6372.1fce | Probably yes | NA^b)^ | Definitely yes (low risk of bias) | NA^b^ | NA^d)^ |
| Poole P, Shulruf B, Rudland J, Wilkinson T. Comparison of UMAT scores and GPA in prediction of performance in medical school: a national study. Med Educ 2012;46:163-171. https://doi.org/10.1111/j.1365-2923.2011.04078.x | Definitely yes (low risk of bias) | NA^b)^ | Definitely yes (low risk of bias) | NA^b^ | NA^d)^ |
| Rauf A, Tayyab A, Masrur A. Relationship between student performances in non-cognitive skills in multiple mini interview and integrated practical examination. J Coll Physicians Surg Pak 2018;28:270-273. https://doi.org/10.29271/jcpsp.2018.04.270 | Probably yes | NA^b)^ | Definitely yes (low risk of bias) | NA^b^ | NA^d)^ |
| Salem RO, Al-Mously N, AlFadil S, Baalash A. Pre-admission criteria and pre-clinical achievement: Can they predict medical students performance in the clinical phase? Med Teach 2016;38 Suppl 1:S26-S30. https://doi.org/10.3109/0142159X.2016.1142511 | Probably yes | NA^b)^ | Definitely yes (low risk of bias) | NA^b^ | NA^d)^ |
| Schneid SD, Kelly CJ, Brandl K. Relationships between preadmission variables and academic outcomes for postbaccalaureate students in medical school. Adv Health Sci Educ Theory Pract 2022;27:1033-1048. https://doi.org/10.1007/s10459-022-10129-3 | Probably yes | NA^b)^ | Definitely yes (low risk of bias) | NA^b^ | NA^d)^ |
| Shulruf B, Poole P, Wang GY, Rudland J, Wilkinson T. How well do selection tools predict performance later in a medical programme? Adv Health Sci Educ Theory Pract 2012;17:615-626. https://doi.org/10.1007/s10459-011-9324-1 | Probably yes | NA^b)^ | Definitely yes (low risk of bias) | NA^b^ | NA^d)^ |
| Simpson PL, Scicluna HA, Jones PD, Cole AM, O'Sullivan AJ, Harris PG, Velan G, McNeil HP. Predictive validity of a new integrated selection process for medical school admission. BMC Med Educ 2014;14:86. https://doi.org/10.1186/1472-6920-14-86 | Probably yes | NA^b)^ | Definitely yes (low risk of bias) | NA^b^ | NA^d)^ |
| Tamimi A, Hassuneh M, Tamimi I, Juweid M, Shibli D, AlMasri B, Tamimi F. Admission criteria and academic performance in medical school. BMC Med Educ 2023;23:273. https://doi.org/10.1186/s12909-023-04251-y | Probably yes | NA^b)^ | Definitely yes (low risk of bias) | NA^b^ | NA^d)^ |
| Tsikas SA. Can selection interviews predict OSCE performance?: evidence from Hannover Medical School. Z Evid Fortbild Qual Gesundhwes 2022;173:85-91. https://doi.org/10.1016/j.zefq.2022.05.008 | Probably yes | NA^b)^ | Definitely yes (low risk of bias) | NA^b^ | NA^d)^ |
| Wilkinson D, Zhang J, Parker M. Predictive validity of the Undergraduate Medicine and Health Sciences Admission Test for medical students’ academic performance. Med J Aust 2011;194:341-344. https://doi.org/10.5694/j.1326-5377.2011.tb03002.x | Probably yes | NA^b)^ | Definitely yes (low risk of bias) | NA^b^ | NA^d)^ |
| Yates J, James D. The value of the UK Clinical Aptitude Test in predicting pre-clinical performance: a prospective cohort study at Nottingham Medical School. BMC Med Educ 2010;10:55. https://doi.org/10.1186/1472-6920-10-55 | Probably yes | NA^b)^ | Definitely yes (low risk of bias) | NA^b^ | NA^d)^ |
| Yates J, James D. The UK Clinical Aptitude Test and clinical course performance at Nottingham: a prospective cohort study. BMC Med Educ 2013;13:32. https://doi.org/10.1186/1472-6920-13-32 | Probably yes | NA^b)^ | Definitely yes (low risk of bias) | NA^b^ | NA^d)^ |
| Nurma Y, Layli I, Kartika P, Syafarinah Nur HA. Admission assessment criteria in predicting students’ academic performance in newly established medical school. Gac Med Caracas 2021;129:S423-S428. https://doi.org/10.47307/GMC.2021.129.s2.21 | Probably yes | NA^b)^ | Definitely yes (low risk of bias) | NA^b^ | NA^d)^ |

| Article | 1. Is the source population representative of the population of interest? | 2. Is the response rate adequate? | 3. Is there little missing data?^a)^ | 4. Is the survey clinically sensible? | 5. Is there any evidence for the reliability and validity of the survey instrument? |
| --- | --- | --- | --- | --- | --- |
| Yusoff MS. The outcomes that an interview-based medical school admission process has on academic performance, psychological health, personality traits, and emotional intelligence. J Taibah Univ Med Sci 2018;13:503-511. https://doi.org/10.1016/j.jtumed.2018.09.003 | Probably yes | NA^b)^ | Probably yes | NA^b^ | NA^d)^ |
| Žuljević MF, Buljan I. Academic and non-academic predictors of academic performance in medical school: an exploratory cohort study. BMC Med Educ 2022;22:366. https://doi.org/10.1186/s12909-022-03436-1 | Probably yes | NA^b)^ | Definitely yes (low risk of bias) | NA^b^ | NA^d)^ |

^a)^This aspect is evaluated by comparing the number of participants whose data were analysed to the original sample size. However, it should be noted that since our meta-analysis extracts the effect of instruments on desired outcomes, this type of missing data does not affect these effect sizes. Therefore, no bias is introduced, regardless of the evaluation. ^b)^Not applicable to studies included in our meta-analysis: as these studies reported results of admission tools and included only participants who were admitted to their respective programs, the response rate is not relevant. ^c)^Not applicable to our meta-analysis: studies included in our meta-analysis reported the results of admission tools already used in their corresponding universities and based outcomes on grade point average, OSCE outcomes, or other prevalent measures, rendering individual face validities irrelevant. ^d)^Not applicable to our meta-analysis: it should be noted that our meta-analysis seeks to determine the effects of admission criteria on outcomes, rather than the validity of admission tools or outcome measures in evaluating student status. Therefore, individual validity or reliability of the tools and measures does not introduce bias to our results.
