# Supplementary material for: Notice of Retraction and Replacement: Insights into undergraduate medical student selection tools: a systematic review and meta-analysis
Source: J Educ Eval Health Prof. 2024 Dec 31;21:41. doi: 10.3352/jeehp.2024.21.41 (PMC11735334; doi:10.3352/jeehp.2024.21.41)
Supplement: Supplementary file 2 — Supplement 2. Replacement article with corrections highlighted. [file jeehp-21-41-suppl2.pdf]

eISSN: 1975-5937
Open Access

Review

J Educ Eval Health Prof 2024;21:22 • https://doi.org/10.3352/jeehp.2024.21.22

Jeehp
Journal of Educational Evaluation for Health Professions

Insights into undergraduate medical student selection tools: a systematic review and meta-analysis

Pin-Hsiang Huang[1,2,3], Arash Arianpoor[1], Silas Taylor[1], Jenzel Gonzales[1], Boaz Shulruf[1,4*]

[1]*Office of Medical Education, Faculty of Medicine & Health, The University of New South Wales, Sydney, Australia*
[2]*Department of Medical Humanities and Medical Education, College of Medicine, National Yang Ming Chiao Tung University, Taipei, Taiwan*
[3]*Division of Infectious Diseases, Department of Medicine, Taipei Veterans General Hospital, Taipei, Taiwan*
[4]*Centre for Medical and Health Sciences Education, University of Auckland, Auckland, New Zealand*

Purpose: Evaluating medical school selection tools is vital for evidence-based student selection. With previous reviews revealing knowledge gaps, this meta-analysis offers insights into the effectiveness of these selection tools.
Methods: A systematic review and meta-analysis were conducted applying the following criteria: peer-reviewed articles available in English, published from 2010 and which include empirical data linking performance in selection tools with assessment and dropout outcomes of undergraduate entry medical programs. Systematic reviews, meta-analyses, general opinion pieces, or commentaries were excluded. Effect sizes (ESs) of the predictability of academic and clinical performance within and by the end of the medicine program were extracted, and the pooled ESs were presented.
Results: Sixty-seven out of 2,212 articles were included, which yielded 237 ESs. Previous academic achievement predicted medical program academic performance (Cohen's d=0.697 in early program; 0.619 in end of program) and clinical exams (0.545 in end of program). Overall aptitude tests predicted academic achievement in both the early and last years (0.550 and 0.371, respectively), as well as the end of program clinical exams (0.448). Within aptitude tests, verbal reasoning and quantitative reasoning best predicted academic achievement in the early program (0.704 and 0.643, respectively). Panel interviews showed no significant effect. However, multiple mini-interviews' effects on early clinical exams and academic performance, as well as situational judgement tests (SJT)'s effect on early academic performance, were statistically significant.
Conclusion: Current evidence suggests that learning outcomes are predicted by previous academic achievement and aptitude tests. The predictive value of SJT and topics such as selection algorithms, features of interview (e.g., content of the questions) and the way the interviewers' reports are used, warrant further research.

Keywords: Academic performance; Algorithms; Aptitude tests; Medical schools; School admission criteria

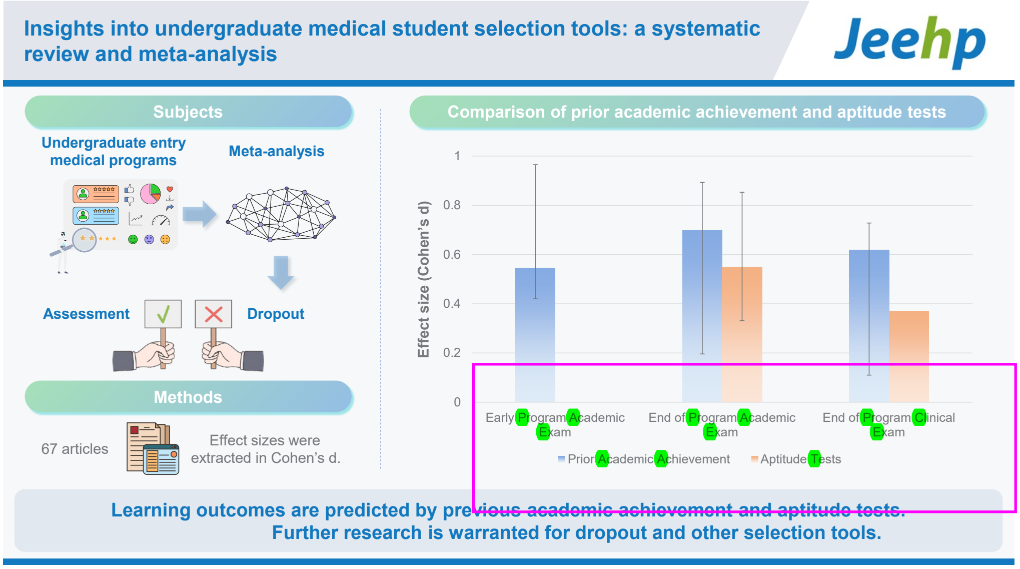

*Corresponding email:
b.shulruf@unsw.edu.au

Editor: Sun Huh, Hallym University, Korea
Received: August 18, 2024
Accepted: September 4, 2024
Published: December 12, 2024
This article is available from: http://jeehp.org

© 2024 Korea Health Personnel Licensing Examination Institute
This is an open-access article distributed under the terms of the Creative Commons Attribution License, which permits unrestricted use, distribution, and reproduction in any medium, provided the original work is properly cited.

Introduction

Background

Continuously and vigorously evaluating medical school selection tools and processes is crucial to ensure that students are selected using the most appropriate and up-to-date empirical evidence available. This is important not only because the initial process largely determines the composition and characteristics of future medical practitioners [1], but also due to the growing complexity of factors being considered in the selection process, including, but not limited to, academic background and aptitudes, social and cultural backgrounds, societal needs, equity, and inclusivity. Currently, there is a plethora of selection tools in the "market" intended to enable selection of candidates, reputedly taking into account a wide range of cognitive and non-cognitive qualities [2,3]. However, whilst there are numerous studies championing the effectiveness (or pointing to the ineffectiveness) of some tools, their predictive validity varies across studies. This variability emphasizes the need for a comprehensive meta-analysis of the efficacy of medical student selection tools in predicting key performance outcomes, to inform all stakeholders.

Reviews of medical school selection tools

Recent systematic and meta-analytic reviews have provided insights into the effectiveness of commonly used medical selection tools. The most prevalent measure—prior academic attainment—has the strongest evidence for use, since it is the most robust predictor across all measures of performance in medical school including attrition. Conversely, the evidence for using personal statements and referee reports is weak [3]. Concerning other tools (e.g., aptitude testing, traditional interviews, situational judgement tests [SJTs], multiple mini-interviews [MMIs] and personality assessments), the evidence regarding their effectiveness is inconclusive [3-9] (Supplement 1). For example, with the more structured MMI being used in place of traditional interviews, numerous reviews have sought to determine effectiveness, since interviewing is a staple in medical selection [10]. Reports about traditional interviews suggest that they yield low reliability [4]. MMIs, on the other hand, have been found to yield higher reliability than panel interviews [3], but could only modestly predict objective structured clinical examination (OSCE) performance, and had weak or no effect with regard to academic performance [4].

Whilst recent reviews have sought to determine the efficacy of these tools, the quality of evaluation of effectiveness of common measurable performance outcomes has been mixed. For example, in relation to SJTs and personality testing, evidence is mostly centered around measuring non-academic value-based attributes [3,5,11] and is largely based on reliability, construct validity, and feasibility measures, whereas predictive validity measures are scarcely reported [3,5]. Whilst evaluating emerging selection tools in this fashion is important, there remains a need to assemble and evaluate the evidence of these tools to predict medical school assessment (e.g., performance in knowledge examinations and clinical assessments) and progression outcomes (e.g., dropout rates, timely completion rates) in order to fully justify their use in selection.

As seen in Supplement 1, a striking feature of the current reviews is a lack of contemporary meta-analytic reviews, with only Goho and Blackman [6] in 2006 and Webster et al. [5] in 2020 conducting meta-analytic studies for interviews and SJTs, respectively. However, on close inspection, the studies included in Goho and Blackman [6] in 2006 had not been selected systematically, raising concerns about the representativeness of the results. This demonstrates the challenge of aspiring to utilize evidence-based, effective selection tools for medical students. Similarly, whilst the systematic reviews (with no meta-analysis) provide some scoping evidence, they lack the empirical measures that meta-analyses provide (Supplement 1).

Noteworthy is that the effectiveness of selection tools in predicting attainment in the medicine program declines over time [12]. Consequently, the expectation of selection tools should be that they are appropriate to predict performance only within the medical program; performance post-medical school should be predicted by medical school outcomes.

Objectives

The objective of the current study was to appraise and map out the empirical evidence available in the literature about the effectiveness of common selection tools in predicting key outcomes in the medicine programs—that is, measured academic and clinical-based performance during the medical program, and overall dropout rates.

Methods

Ethics statement

As this study is based solely on existing literature, it does not necessitate approval from the institutional review board or the acquisition of informed consent.

Study design

The presentation of this systematic review and meta-analysis adhered to the guidelines outlined in the Preferred Reporting

Items for Systematic Reviews and Meta-Analysis [13].

Eligibility criteria

Titles and keywords of all articles generated by the search were reviewed to remove clearly irrelevant and duplicate articles. Next, abstracts of the remaining articles were read to eliminate irrelevant articles and highlight potentially relevant articles. Finally, remaining articles were reviewed until authors agreed on the articles to be included in the data extraction based on the following inclusion criteria: (1) articles including a measure of medical school performance which was related to performance in a selection tool; (2) the participants were attempting selection at an undergraduate medical program; (3) articles were peer-reviewed and available in English; and (4) articles were published from 2010 onwards. The exclusion criterion applied to articles as a systematic review, meta-analysis, general opinion piece, or a commentary.

Information sources and search strategy

The search of the literature was completed in July 2024 using the SCOPUS database, which provides comprehensive coverage of health and medical science [14]. Results were limited to studies published from 2010 onwards (Supplement 2).

- #1 Five selection tools: Academic Achievement, Aptitude Tests, MMI and Interviews, Situational Judgement Tests, Cognitive Tests (please see Supplement 2 for full search terms for each tool)
- #2 Add specification of prediction or selection tools for admission
- #3 Target aim for the selection process of medical school admission
- #4 Outcome variables for academic achievement, clinical assessment, and dropout from medicine: ("academic record" OR "academic attainment" OR "academic performance" OR "gpa" OR "clinical assessment" OR "osce" OR "grade point average" OR "fail" OR "performance" OR "skills assessment" OR "assessment" OR "competency" OR "pbl" OR "completion")
- #5 Combination of each #1 AND #2 AND #3 AND #4: total five search results (one result for each selection tool, and five tools were included in #1)

Selection process

From initial search results, articles were removed due to irrelevance, duplication, or insufficient data to be extracted for effective sizes. All search steps and processes were confirmed by 3 authors (P.H.H., J.G., and B.S.). Results of the 5 searches were filtered and extracted by 2 authors (A.A. and J.G.) with confirmation with another (P.H.H.), and disagreement was resolved by corresponding author (B.S.) where necessary.

Data collection process, data items, effect measures, synthesis methods, and certainty assessment

Author, year of publication, country, selection tools, outcomes (clinical and academic assessment results and dropout rate, and the former 2 were further split to first known results and final year results), and sample size and analysis were recorded for each study and analysis. Correlations, regressions, odds ratios, t-tests, and chi-square were extracted as appropriate results and used to calculate effect sizes. The selection tool as well as the outcome were noted and later grouped to form the basis for the meta-analysis. The process was done by 2 authors (J.G. and A.A.) and confirmed by another (P.H.H.). Effect sizes were initially converted into Fisher's Z (Zr), Cohen's d (d), and odds ratios. Once the effect sizes were collated, they were converted to Cohen's d to run the meta-analysis with a common effect size. After effect sizes were calculated, a meta-analysis was performed for each combination of selection tool and outcomes where there were more than 2 effect sizes available for the grouping. Due to the heterogeneous nature of the application of selection tools across studies (i.e., different countries, weightings of selection criteria, and medical programs) a random-effects meta-analysis was conducted [15]. This was done using Microsoft Excel (Microsoft Corp.), and confidence interval (CI) for each effect size was calculated to assess a level of certainty. All analyses have been double-checked using IBM SPSS Statistics ver. 28.0.1.0 (142) (IBM Corp.) and Jamovi ver. 2.6.13 (The Jamovi Project), incorporating the MAJOR (Meta-Analysis for Jamovi) 1.2.4 package, with files filtered for output (see Dataset 1 for more details).

Study risk of bias assessment and reporting bias assessment

The Risk of Bias Instrument (CLARITY group, McMaster University) is utilized to appraise the risk of bias in individual studies [16].

Results

Study selection

The initial search on the SCOPUS database identified 2,212 articles including duplicates. Titles were reviewed to remove clearly irrelevant articles and duplicates, leaving 510 articles (Fig. 1). Abstracts and full-text of remaining studies were then reviewed in accordance with inclusion and exclusion criteria, further removing

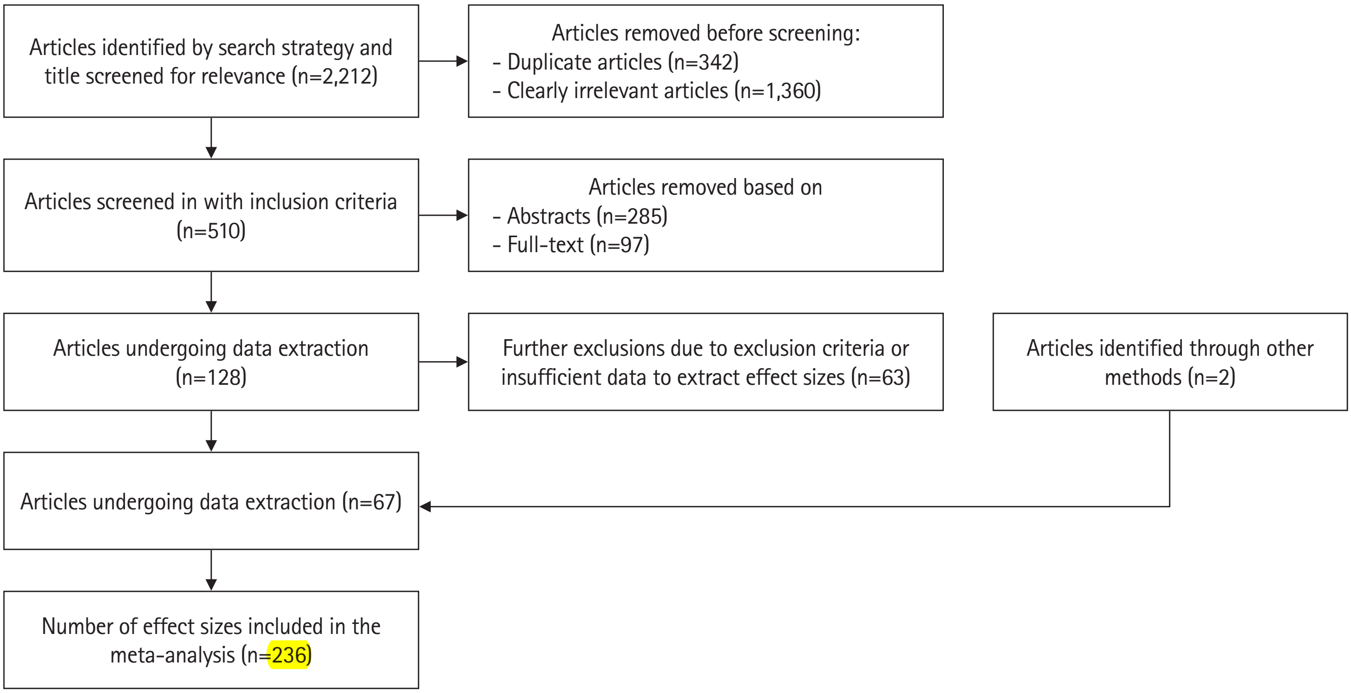

Fig. 1. Flow chart of included studies and derived effect sizes.

Table 1. Number of articles and effect sizes in each category of selection tool

Selection tool	Example selection tool	Articles	Effect sizes
Prior academic achievement	High school grade point average; Australian Tertiary Admission Rank	47	94
Aptitude testing	UK Clinical Aptitude Test overall	27	97
MMI	MMI	9	12
Panel interview	Semi-structured interview	12	20
Situational judgement test	Computer-based Assessment for Sampling Personal characteristics	3	10
Personality testing	Personality Qualities Assessment	3	3

Some articles included more than one selection tool category and were independently included in this table.
MMI, multiple mini-interview.

285 articles based on their abstracts and 97 articles from full-text. An additional 2 articles were identified from reference sections. This initially left 130 articles for data extraction. However, upon inspection of individual study data, a further 63 studies were excluded due to exclusion criteria or insufficient data allowing for effect sizes to be extracted in line with this study's objectives. Consequently, 67 articles with at least 1 effect size extracted were included in the meta-analysis, with a total of 237 usable effect sizes.

Study characteristics

Studies provided data on predictions of various outcomes of student performance within medicine programs by selection tools. Of the 67 studies included in meta-analysis, there were up to 12 effect sizes per study. These studies were conducted across 6 continents.

Predictor variables were selection tools used by medical school programs (Table 1). These included measures of prior academic achievement (e.g., high school grade point average and high school exams), aptitude tests (e.g., UK Clinical Aptitude Test and Undergraduate Medical Admissions Test), panel interviews and MMIs, SJTs, and personality assessments (e.g., Personal Qualities Assessment).

Outcome variables were medical school academic results (split into early program and end of program results), OSCE/objective structured long case examination record (OSLER)/clinical exams, and progression outcomes (i.e., dropout and repetition). OSLER is included in this analysis because some medical schools favor this clinical assessment, as it has been shown to have good validity and reliability when well-planned and executed [17].

Table 2. Summary of meta-analysis results

Selection tools	OSCE/OSLER/clinical exams		Academic results		Dropout
	Early program	End of program	Early program	End of program	
Academic achievement	**0.238 (0.070 to 0.406) [9 (9)]**	**0.545 (0.235 to 0.855) [8 (7)]**	**0.697 (0.533 to 0.861) [40 (29)]**	**0.619 (0.533 to 0.705) [34 (20)]**	0.205 (–0.162 to 0.572) [3 (3)]
Aptitude tests					
Overall	0.106 (–0.059 to 0.270) [6 (5)]	**0.448 (0.019 to 0.877) [6 (5)]**	**0.550 (0.390 to 0.710) [20 (16)]**	**0.371 (0.278 to 0.463) [13 (11)]**	0.082 (–0.395 to 0.559) [4 (4)]
Abstract reasoning	*Nil ES*	**0.221 (0.016 to 0.427) [4 (4)]**	**0.167 (0.009 to 0.324) [8 (6)]**	**0.211 (0.117 to 0.305) [6 (6)]**	*Nil ES*
Interpersonal reasoning	*Nil ES*	*0.473 (0.172 to 0.773) [1 (1)]*	**0.276 (0.056 to 0.496) [7 (5)]**	*0.074 (–0.058 to 0.206) [2 (2)]*	*Nil ES*
Verbal reasoning	*Nil ES*	**0.298 (0.004 to 0.592) [4 (4)]**	**0.704 (0.471 to 0.938) [4 (3)]**	**0.305 (0.203 to 0.407) [3 (3)]**	*Nil ES*
Quantitative reasoning	*Nil ES*	0.216 (–0.030 to 0.462) [3 (3)]	**0.643 (0.328 to 0.958) [4 (3)]**	**0.144 (0.091 to 0.196) [3 (3)]**	*Nil ES*
Interviews					
MMI	**0.417 (0.092 to 0.743) [6 (5)]**	*Nil ES*	**0.195 (0.019 to 0.372) [4 (3)]**	*0.229 (0.081 to 0.377) [1 (1)]*	*–0.014 (–0.193 to 0.165) [1(1)]*
Panel	0.091 (–0.110 to 0.293) [3 (3)]	0.372 (–0.132 to 0.875) [5 (4)]	0.121 (–0.185 to 0.426) [6 (6)]	0.135 (–0.018 to 0.288) [5 (5)]	*0.459 (0.235 to 0.683) [1 (1)]*
Situational judgement tests	*Nil ES*	*Nil ES*	**0.170 (0.032 to 0.308) [8 (2)]**	*0.216 (0.118 to 0.313) [2 (2)]*	*Nil ES*
Personality tests					
PQA	*0.004 (–0.103 to 0.111) [1 (1)]*	*0.172 (–0.001 to 0.345) [1 (1)]*	*Nil ES*	*Nil ES*	*Nil ES*
MMPI	*Nil ES*	*Nil ES*	*0.131 (–0.067 to 0.329) [1 (1)]*	*Nil ES*	*Nil ES*

Values are presented as ES (95% confidence interval) [number of ESs (number of included articles)]. Included articles may report more than one ES within the same cell if different cohorts were studied. Statistically significant ESs are highlighted in bold. Results are italicized where number of ESs are less than 3.
OSCE, objective structured clinical exam; OSLER, objective structured long examination record; ES, effect size; MMI, multiple mini interview; PQA, Personal Qualities Assessment; MMPI, Minnesota Multiphasic Personality Inventory.

Results of the meta-analysis

Results of the random-effects analyses are shown in Table 2 (Supplement 3 for reference articles). Results extracted from only 1 or 2 effect sizes are still shown in the table, but are not discussed due to insufficient evidence.

Prior academic achievement

Findings show that prior academic achievement best predicts academic results for early program and end of program time points with effect sizes of 0.697 (95% CI, 0.533 to 0.861) and 0.619 (95% CI, 0.533 to 0.705), respectively, as well as performance on end of program OSCE/OSLER/clinical exams (0.545; 95% CI, 0.235 to 0.855). The effect size of prior academic achievement on early program OSCE/OSLER/clinical exams was 0.238 (95% CI, 0.070 to 0.406).

Aptitude testing

Aptitude tests overall best predict early program academic results (~~0.550,~~ 95% CI, 0.390 to 0.710) and both end of program academic results (~~0.371,~~ 95% CI, 0.278 to 0.463) and OSCE/OSLER/clinical exams (0.448; 95% CI, 0.019 to 0.877). Effect size for early program OSCE/OSLER/clinical exams was insignificant (0.106; 95% CI, -0.059 to 0.270).

With aptitude test subtest domains, abstract reasoning and verbal reasoning had effect sizes of 0.211 (95% CI, 0.117 to 0.305) and 0.305 (95% CI, 0.203 to 0.407), respectively for end of program academic results, and 0.221 (95% CI, 0.016 to 0.427) and 0.298 (95% CI, 0.004 to 0.592), respectively for end of program OSCE/OSLER/clinical exams. Moreover, verbal reasoning, quantitative reasoning, and interpersonal reasoning had effect sizes of 0.704 (95% CI, 0.471 to 0.938), 0.643 (95% CI, 0.328 to 0.958), and 0.276 (95% CI, 0.056 to 0.496), respectively for early program academic results. Although the effect of abstract reasoning for early program academic results was less than 0.2, it reached statistical significance (0.167; 95% CI, 0.009 to 0.324).

Quantitative reasoning had an effect size of 0.216 for end of

program OSCE/OSLER/clinical exams, but the 95% CIs intersected zero. In contrast, while its effect size for end of program academic results was less than 0.2, it was statistically significant (0.144; 95% CI, 0.091 to 0.196).

Interviews

The effect sizes of MMI's were 0.417 (95% CI, 0.092 to 0.743) for early program OSCE/OSLER/clinical exams and 0.195 (95% CI, 0.019 to 0.372) for early program academic results. There were insufficient effect sizes (less than 3) for all other outcomes.

Panel interviews had effect sizes smaller than 0.2 for early and end of program academic results, and an effect size of 0.372 for end of program OSCE/OSLER/clinical exams. However, these 3 effect sizes had 95% CIs intersecting zero.

SJT and personality testing

While SJT had an effect size of less than 0.2 on early program academic results, its effect reached statistical significance (0.170; 95% CI, 0.032 to 0.308). For personality tests, the extracted effect sizes were insufficient to conduct a meta-analysis for any of the outcomes of interest.

Risk of bias in studies, and reporting biases

In our 67 studies, the risk of bias was low regarding representativeness (11 definitely yes for low risk, 56 probably yes) and little missing data (31 definitely yes, 33 probably yes, 1 probably no, 2 definitely no). Due to the nature of student selection tool meta-analysis which aimed to evaluate the predictive value of each tool, the other 3 risks of bias were not applicable, namely, response rates, surface validity of surveys, and evidence for reliability and validity of surveys (see Supplement 4 for more details).

Discussion

Interpretation

All studies examined how selection tools predict undergraduate medical school outcomes. When examining the tools with medium effect sizes ($d \geq 0.5$) [18], only prior academic achievement had a medium effect size to predict end of program OSCE results (0.545, $P < 0.05$). As for academic results, selection tools with predictive effect sizes larger than 0.5 were prior achievement (0.697, $P < 0.05$ in early program; 0.619, $P < 0.05$ in end of program) and overall aptitude testing (0.550, $P < 0.05$ in early program). Additionally, the effects of 2 subcategories of aptitude testing, verbal reasoning (0.704, $P < 0.05$) and quantitative reasoning (0.643, $P < 0.05$), on early program academic results were notable. Overall, these results reflect the common view of the efficacy of previous academic achievement as the main tool for predicting performance in a medical program, supporting the use of this tool in medical student selection [19-21].

OSCEs test a set of clinical skills in multiple stations, where students are expected to demonstrate their ability to perform history taking, physical examination, subsequent management plans, explanation of diagnosis, and procedural skills. Many OSCEs utilize combinations of checklists and global ratings [22,23]. Although OSCE is taken in the form of multiple stations, students may prepare for OSCEs by memorizing textbook materials for the checklists. Therefore, it is reasonable that prior academic achievement, which reflects students' abilities to comprehend and memorize knowledge, yields medium effect size in predicting OSCE results since the cognitive skills used are similar.

In this respect, when examining the remaining meta-analysis results with the number of effect sizes larger than 2 (i.e., ignoring the numbers italicized in Table 2 due to low evidence), there was no tool other than previous academic achievement demonstrating a large effect size ($d > 0.5$) in predicting clinical performance outcomes. However, it should be noted that the effects of overall aptitude testing—as well as its subcategories of abstract reasoning and verbal reasoning—and MMI were statistically significant (all $P < 0.05$). Perhaps verbal reasoning may interact with student performance in certain stations related to patient communication skills [24], and the association between MMI and OSCE could be partially explained by the similarity in conduct of multiple stations with the grading system by checklist and global ratings [25]. Although these findings support the use of MMI as a valid selection tool, the evidence as to whether the predictability is related more to the form of the assessment (multiple short sessions), or to the skills assessed in both MMI and OSCE, is yet to be presented. It is possible that the multiple short sessions format suits applicants (who later become students) who are able to generate good first impressions. Thus, they are more advantaged than others in both MMI and OSCE, since they have multiple chances to utilize the halo effect on the interviewers/assessors [26,27]. Note that multiple data points increase the statistical reliability of the final results, yet that cannot mitigate the inherent bias generated by the halo effect. More research is needed to better understand the strengths and limitations of the MMI as a valid selection tool.

The results provide important insights into the associations between the selection tools and performance within the medicine program. First, previous academic achievement and aptitude tests predicted academic outcomes with medium effect sizes ($d > 0.5$) and as expected, due to the length of the program (5–7 years), the effect drops somewhat from the early program to the end of the program. In addition, the specific components within the aptitude

tests demonstrate that abstract, verbal, and quantitative reasoning are important qualities for success in medical school throughout the program, both in the early program and later clinical stages of curricula. On the other hand, quantitative reasoning is important in the early program, whereas abstract and verbal reasoning becomes important at the clinical stage, when significant integration and presentation of clinical, biomedical, and psychosocial information is required. Furthermore, it appears that interpersonal reasoning skills have small, yet statistically significant predictive value for early academic performance, and more evidence is needed to understand its predictive value for clinical performance and end of program academic performance in medicine. Further insights emerge from these findings. First, when medical schools use aptitude tests, they should consider each component separately rather than relying on the overall score. More specifically, it is suggested that different weights should be allocated to each of the components based on the specific curriculum emphases and the known predictive power of each of these components. Second, measuring interpersonal attributes using tools that do not directly engage interpersonal interaction may not be optimal, as none of the written tests (including SJT and personality tests) measuring interpersonal interaction yielded large effect sizes ($d > 0.5$) in predicting any such related outcomes. Although MMI predicted early clinical and academic performances and SJT predicted early academic performances with small effect sizes, more evidence is needed to determine whether they could predict clinical or academic performance at the end of medicine program.

In light of these findings, the remaining question is where to from here? Apart from the known significance of previous academic achievement, the results suggest a potential direction for the use of tools measuring interpersonal interaction. The MMI is the most promising tool for predicting performance in clinical examination assessments in the early years of the medicine program, whereas the panel interviews appear to predict such performance at the end of the program. Yet, the papers reviewed do not provide detailed information on the content and any other specifications of the interviews/MMI. Thus, it is possible that the format of the interviews (i.e., panel versus MMI) is not as important as some have suggested [28]. Instead, the specifics of the interviews—such as what is asked, how questions are phrased, the characteristics of the interviewers, how the data are computed and used, and similar considerations—should be prioritized while selection interviews are employed [26,27,29].

The third insight coming out of this meta-analysis is about what is missing, that is, the evidence that could not be found in the literature. It is quite striking to see that very little is known about the prediction of (timely) completion (or dropout) by the selection tools. Obviously, this topic has not been sufficiently investigated despite timely completion being the most critical outcome of the medicine program [19]. Research into this topic is urgently required, particularly in light of the growing trend to shift assessment in the medicine program from graded to ungraded [30,31], which limits the ability to undertake meaningful statistical analysis associating selection tools with performance in the program. Also of note is the scarcity of empirical data about the association between SJT (with only 7 effect sizes from a single study and just one from another) and/or personality tests (with only one effect size) and performance in the medicine program. Again, in the light of the growing use of these tools for selection, there is a need to undertake more empirical research to identify what these tools predict and how best they should be utilized.

Comparison with previous studies

Our study showed similar results compared to 2 previous systematic reviews for aptitude tests. One review showed 14 papers with weak relationship to the assessment outcomes whereas the other 4 papers showed moderate relationship [8]. Although another review showed 70% of data points with insignificant predictive value, many of the significant values predicted early academic achievement [9]. With extensive search and calculation, our meta-analysis concluded that overall aptitude tests and all its sub-categories (abstract reasoning, interpersonal reasoning, verbal reasoning, and quantitative reasoning) could predict academic achievement.

The only meta-analysis in recent years by Webster et al. [5] in 2020 targeted SJT and showed a significant moderate pooled effect size (Pearson's correlation coefficient = 0.32). However, their meta-analysis, incorporating 26 studies, consisted of numerous outcomes including but not limited to simulation, supervisor and tutor ratings, MMI, case logs, and other forms of SJT, while the differences of the effect on specific outcomes has not been investigated [5]. Therefore, it may be difficult to indicate how SJT impacts on clinical performance, academic performance, and dropout rate from their pooled results and the data available to date.

Limitation

As the first known meta-analysis of its kind that comprehensively incorporates and compares different selection tools by effect sizes of their predictive power, this study has 2 limitations. The first limitation is the remarkable scarcity of empirical data particularly related to interviews, SJT, and personality test. This is a limiting factor in terms of extracting effect sizes, yet, as mentioned above, knowing what is unknown is important and can provide clear direction for future research.

The second limitation is that the outcome variables could only include the students who were successfully enrolled in medicine, while data of those who were not enrolled could not be measured. This may impact the primary data since there are no control groups. However, this limitation could not be resolved due to the nature of student selection process and data availability.

Implications

Interviews, either panel or MMI, have the potential to predict academic and clinical outcomes at the end of medicine program, yet more studies are required to empirically establish this finding, particularly looking at the content of the interview questions. SJT and personality tests also may have the potential to predict performance in the medicine program; however, the evidence is too scarce and too weak at this stage to suggest conclusive recommendation. In addition, further research is required to investigate the predictability of medicine program outcomes by specific components (domains) of the aptitude test.

The impact of the selection algorithm on the outcome has been overlooked in the literature and consequently was not included in this meta-analysis. Two recent studies demonstrated that a non-compensatory selection algorithm yields higher predictive power over compensatory selection algorithm when the very same selection tools are used [20,29]. Unfortunately, no other studies looking into this topic were found. Therefore, it is suggested that further research into the efficacy of selection algorithms is undertaken to further improve selection to medical schools.

Conclusion

Previous academic achievement as well as aptitude tests are valid tools for predicting performance in the medicine program. There is a need for further research into interviews, SJTs, and personality tests to determine their predictability of desired outcomes. Medical school committees tasked to address medical student selection may consider these tools and evidence when designing the selection process and associated algorithms.

ORCID

Pin-Hsiang Huang: https://orcid.org/0000-0002-6101-0080; Arash Arianpoor: https://orcid.org/0000-0001-5646-2333; Silas Taylor: https://orcid.org/0000-0003-1992-8485; Jenzel Gonzales: https://orcid.org/0009-0005-9606-3659; Boaz Shulruf: https://orcid.org/0000-0003-3644-727X

Authors' contributions

Conceptualization: PHH, BS. Data curation: PHH, JG, AA, BS. Methodology/formal analysis/validation: PHH, AA, JG, ST, BS. Project administration: BS. Funding acquisition: BS. Writing–original draft: PHH, JG. Writing–review & editing: PHH, JG, AA, ST, BS.

Conflict of interest

Boaz Shulruf has been an associate editor of the *Journal of Educational Evaluation for Health Professions* since 2017 but had no role in the decision to publish this review. Except for that, no other potential conflict of interest relevant to this article was reported.

Funding

This research was funded by the UCAT ANZ Consortium, Australia. The funders had no role in study design, data collection and analysis, decision to publish, or preparation of the manuscript.

Data availability

Data files are available from Harvard Dataverse: ~~
Dataset 1. Extracted data is provided in the format of excel file.

Acknowledgments

None.

Supplementary materials

Supplementary files are available from Harvard Dataverse: https://doi.org/10.7910/DVN/OVVPST
Supplement 1. Summary of previous systematic reviews and meta-analyses of medical student selection tools.
Supplement 2. Search scripts.
Supplement 3. References of articles included in the meta-analysis.
Supplement 4. Bias appraisal.
Supplement 5. Audio recording of the abstract.

References

1. Razack S. 'Meritocracy' and 'fairness' in medical student selection: comparing UK and Australia. Med Educ 2021;55:772-774. https://doi.org/10.1111/medu.14525

2. Kreiter C, O'Shea M, Bruen C, Murphy P, Pawlikowska T. A meta-analytic perspective on the valid use of subjective human judgement to make medical school admission decisions. Med Educ Online 2018;23:1522225. https://doi.org/10.1080/10872981.2018.1522225
3. Patterson F, Knight A, Dowell J, Nicholson S, Cousans F, Cleland J. How effective are selection methods in medical education?: a systematic review. Med Educ 2016;50:36-60. https://doi.org/10.1111/medu.12817
4. Lin JC, Lokhande A, Margo CE, Greenberg PB. Best practices for interviewing applicants for medical school admissions: a systematic review. Perspect Med Educ 2022;11:239-246. https://doi.org/10.1007/s40037-022-00726-8
5. Webster ES, Paton LW, Crampton PE, Tiffin PA. Situational judgement test validity for selection: a systematic review and meta-analysis. Med Educ 2020;54:888-902. https://doi.org/10.1111/medu.14201
6. Goho J, Blackman A. The effectiveness of academic admission interviews: an exploratory meta-analysis. Med Teach 2006;28:335-340. https://doi.org/10.1080/01421590600603418
7. Callwood A, Jeevaratnam K, Kotronoulas G, Schneider A, Lewis L, Nadarajah VD. Personal domains assessed in multiple mini interviews (MMIs) for healthcare student selection: a narrative synthesis systematic review. Nurse Educ Today 2018;64:56-64. https://doi.org/10.1016/j.nedt.2018.01.016
8. Greatrix R, Nicholson S, Anderson S. Does the UKCAT predict performance in medical and dental school?: a systematic review. BMJ Open 2021;11:e040128. https://doi.org/10.1136/bmjopen-2020-040128
9. Bala L, Pedder S, Sam AH, Brown C. Assessing the predictive validity of the UCAT: a systematic review and narrative synthesis. Med Teach 2022;44:401-409. https://doi.org/10.1080/0142159X.2021.1998401
10. Ali S, Sadiq Hashmi MS, Umair M, Beg MA, Huda N. Multiple mini-interviews: current perspectives on utility and limitations. Adv Med Educ Pract 2019;10:1031-1038. https://doi.org/10.2147/AMEP.S181332
11. Patterson F, Zibarras L, Ashworth V. Situational judgement tests in medical education and training: research, theory and practice: AMEE guide no. 100. Med Teach 2016;38:3-17. https://doi.org/10.3109/0142159X.2015.1072619
12. Simpson PL, Scicluna HA, Jones PD, Cole AM, O'Sullivan AJ, Harris PG, Velan G, McNeil HP. Predictive validity of a new integrated selection process for medical school admission. BMC Med Educ 2014;14:86. https://doi.org/10.1186/1472-6920-14-86
13. Page MJ, McKenzie JE, Bossuyt PM, Boutron I, Hoffmann TC, Mulrow CD, Shamseer L, Tetzlaff JM, Akl EA, Brennan SE, Chou R, Glanville J, Grimshaw JM, Hrobjartsson A, Lalu MM, Li T, Loder EW, Mayo-Wilson E, McDonald S, McGuinness LA, Stewart LA, Thomas J, Tricco AC, Welch VA, Whiting P, Moher D. The PRISMA 2020 statement: an updated guideline for reporting systematic reviews. BMJ 2021;372:n71. https://doi.org/10.1136/bmj.n71
14. Zhu J, Liu W. A tale of two databases: the use of Web of Science and Scopus in academic papers. Scientometrics 2020;123:321-335. https://doi.org/10.1007/s11192-020-03387-8
15. Tufanaru C, Munn Z, Stephenson M, Aromataris E. Fixed or random effects meta-analysis?: common methodological issues in systematic reviews of effectiveness. Int J Evid Based Healthc 2015;13:196-207. https://doi.org/10.1097/XEB.0000000000000065
16. Agarwal A, Guyatt G, Busse J. Methods commentary: risk of bias in cross-sectional surveys of attitudes and practices. McMaster University; 2019
17. Luder A. Assessment of clinical education. In: Dickman N, Schuster B, editors. Active education for future doctors. Springer; 2020. p. 67-78. https://doi.org/10.1007/978-3-030-41780-2_6
18. Lovakov A, Agadullina ER. Empirically derived guidelines for effect size interpretation in social psychology. Eur J Soc Psychol 2021;51:485-504. https://doi.org/10.1002/ejsp.2752
19. Shulruf B, Bagg W, Begun M, Hay M, Lichtwark I, Turnock A, Warnecke E, Wilkinson TJ, Poole PJ. The efficacy of medical student selection tools in Australia and New Zealand. Med J Aust 2018;208:214-218. https://doi.org/10.5694/mja17.00400
20. Shulruf B, O'Sullivan A, Velan G. Selecting top candidates for medical school selection interviews: a non-compensatory approach. BMC Med Educ 2020;20:113. https://doi.org/10.1186/s12909-020-02031-6
21. Sladek RM, Bond MJ, Frost LK, Prior KN. Predicting success in medical school: a longitudinal study of common Australian student selection tools. BMC Med Educ 2016;16:187. https://doi.org/10.1186/s12909-016-0692-3
22. Hurley KF, Giffin NA, Stewart SA, Bullock GB. Probing the effect of OSCE checklist length on inter-observer reliability and observer accuracy. Med Educ Online 2015;20:29242. https://doi.org/10.3402/meo.v20.29242
23. Sim JH, Aziz YF, Vijayanantha A, Mansor A, Vadivelu J, Hassan H. A closer look at checklist scoring and global rating for four OSCE stations: do the scores correlate well? Educ Med J 2015;7:e39-e44. https://doi.org/10.5959/eimj.v7i2.341
24. O'Sullivan DM, Moran J, Corcoran P, O'Flynn S, O'Tuathaigh

C, O'Sullivan AM. Medical school selection criteria as predictors of medical student empathy: a cross-sectional study of medical students, Ireland. BMJ Open 2017;7:e016076. https://doi.org/10.1136/bmjopen-2017-016076
25. Kumar N, Bhardwaj S, Rahman E. Multiple mini-interview as a predictor of performance in the objective structured clinical examination among Physician Associates in the United Kingdom: a cohort study. Adv Med Educ Pract 2018;9:239-245. https://doi.org/10.2147/AMEP.S159412
26. Reiter H, Eva K. Vive la Différence: the freedom and inherent responsibilities when designing and implementing multiple mini-interviews. Acad Med 2018;93:969-971. https://doi.org/10.1097/ACM.0000000000002042
27. Towaij C, Gawad N, Alibhai K, Doan D, Raiche I. Trust me, I know them: assessing interpersonal bias in surgery residency interviews. J Grad Med Educ 2022;14:289-294. https://doi.org/10.4300/JGME-D-21-00882.1
28. Griffin B, Auton J, Duvivier R, Shulruf B, Hu W. Multiple mini interviews: revealing similarities across institutions. BMC Med Educ 2018;18:190. https://doi.org/10.1186/s12909-018-1298-8
29. Shulruf B, Velan GM, Kennedy SE. Medical student selection process enhanced by improving selection algorithms and changing the focus of interviews in Australia: a descriptive study. J Educ Eval Health Prof 2022;19:31. https://doi.org/10.3352/jeehp.2022.19.31
30. Dolan BM, Arnold J, Green MM. Establishing trust when assessing learners: barriers and opportunities. Acad Med 2019;94:1851-1853. https://doi.org/10.1097/ACM.0000000000002982
31. Moltner A, Timbil S, Junger J. The reliability of the pass/fail decision for assessments comprised of multiple components. GMS Z Med Ausbild 2015;32:DOC42. https://doi.org/10.3205/zma000984
